# Human menstrual blood-derived stem cells mitigate bleomycin-induced pulmonary fibrosis through anti-apoptosis and anti-inflammatory effects

**DOI:** 10.1186/s13287-020-01926-x

**Published:** 2020-11-11

**Authors:** Xin Chen, Yi Wu, Yanling Wang, Lijun Chen, Wendi Zheng, Sining Zhou, Huikang Xu, Yifei Li, Li Yuan, Charlie Xiang

**Affiliations:** 1 State Key Laboratory for Diagnosis and Treatment of Infectious Diseases and Collaborative Innovation Center for Diagnosis and Treatment of Infectious Diseases, The First Affiliated Hospital, School of Medicine, Zhejiang University, Hangzhou, 310027 People’s Republic of China; 2 Innovative Precision Medicine (IPM) Group, Hangzhou, 311215 China

**Keywords:** Menstrual blood-derived mesenchymal stem cells, Bleomycin, Pulmonary fibrosis, Stem cell transplantation therapy, Apoptosis, Inflammation

## Abstract

**Background:**

Idiopathic pulmonary fibrosis is a kind of diffuse interstitial lung disease, the pathogenesis of which is unclear, and there is currently a lack of good treatment to improve the survival rate. Human menstrual blood-derived mesenchymal stem cells (MenSCs) have shown great potential in regenerative medicine. This study aimed to explore the therapeutic potential of MenSCs for bleomycin-induced pulmonary fibrosis.

**Methods:**

We investigated the transplantation of MenSCs in a pulmonary fibrosis mouse model induced by BLM. Mouse was divided into three groups: control group, BLM group, MenSC group. Twenty-one days after MenSC transplantation, we examined collagen content, pathological, fibrosis area in the lung tissue, and the level of inflammatory factors of serum. RNA sequence was used to examine the differential expressed gene between three groups. Transwell coculture experiments were further used to examine the function of MenSCs to MLE-12 cells and mouse lung fibroblasts (MLFs) in vitro.

**Results:**

We observed that transplantation of MenSCs significantly improves pulmonary fibrosis mouse through evaluations of pathological lesions, collagen deposition, and inflammation. Transwell coculturing experiments showed that MenSCs suppress the proliferation and the differentiation of MLFs and inhibit the apoptosis of MLE-12 cells. Furthermore, antibody array results demonstrated that MenSCs inhibit the apoptosis of MLE-12 cells by suppressing the expression of inflammatory-related cytokines, including RANTES, Eotaxin, GM-CSF, MIP-1γ, MCP-5, CCL1, and GITR.

**Conclusions:**

Collectively, our results suggested MenSCs have a great potential in the treatment of pulmonary fibrosis, and cytokines revealed in antibody array are expected to become the target of future therapy of MenSCs in clinical treatment of pulmonary fibrosis.

## Background

Pulmonary fibrosis is a fatal interstitial lung disease which is characterized by fibroblast proliferation and extracellular matrix formation, and accompanied by inflammatory lesions and structural damage. Many patients are often diagnosed in the end stage due to misdiagnosis in the early stage; thus, it is difficult to administer early treatment, and most patients survive less than 5 years [1–3]. Currently, lung transplantation is the optimal option to improve the survival rate and quality of life of pulmonary fibrosis patients. However, this option is limited due to a lack of donors [4]. Thus, there is an urgent need to find alternative therapeutic methods for pulmonary fibrosis. With the development of cell-based therapy, stem cells have become a promising choice for treating a series of diseases.

Mesenchymal stem cells (MSCs), one of the most popular adult stem cells, are derived from the mesoderm and can differentiate into different cell lines including osteoblasts, adipocytes, and chondrocytes [5]. In recent decades, MSCs have been thought to be an alternative treatment for pulmonary fibrosis due to their therapeutic potential [6–9]. MSCs can home to the injury site, reduce the deposition of collagen and inhibit pro-inflammatory factors [10]. Bone marrow (BM)-MSC, adipose tissue-MSC, and umbilical cord (UC)-MSC transplantations improve the symptoms of pulmonary fibrosis [8, 9, 11]. However, invasive access and a lack of sources for these cells have limited their application, especially when a large number of primary cultured MSCs are needed. Therefore, novel sources of MSCs are urgently needed to solve this problem.

Currently, human menstrual blood-derived stem cells (MenSCs) are isolated from women’s menstrual blood in menstruation [12, 13]. Since MenSCs were discovered by Meng et al. in 2007 [13], MenSCs have gained increasing attention. Compared with other MSCs, MenSCs have a high proliferation rate and wide range of sources, and accessing them is simple and painless [14–17]. In addition, MenSCs have low immunogenicity because of the low expression of major histocompatibility complex class II (MHC-II), which can be expanded through more than 20 passages without genetic mutation [18–20]. Furthermore, our group has reported the therapeutic effects of MenSCs in different animal models, which include liver fibrosis and acute lung injury [21, 22]. Based on our previous research, we further explored the impact of MenSCs on bleomycin (BLM)-induced pulmonary fibrosis and the underlying mechanisms.

In the present study, we aimed to assess the therapeutic effect of MenSCs in mice with bleomycin (BLM)-induced pulmonary fibrosis. We further used transwell coculture assays to elucidate the underlying mechanism of MenSC activity in vitro. Our research contributes to an understanding of the function of MenSCs in pulmonary fibrosis and suggests that MenSCs are promising tool for treating severe pulmonary disease in future clinical research.

## Methods

### Animals and cells

Male C57BL/6J mice (6–8 weeks old) were purchased from the Slac Laboratory Animal Corporation (Shanghai, China) and housed at the Laboratory Animal Center of Zhejiang University with access to plenty of food and water. All animal experiments were approved by the tab of Animal Experiment Ethical Inspection of the Affiliated Hospital of Medicine, Zhejiang University, the approval number is 2017-574.

MenSCs were isolated and cultured as previously described [19, 23]. Briefly, menstrual blood samples were collected with a Divacup (Kitchener, ON, Canada) from healthy women who were in menstruation before signing an informed consent; this was approved by Approved Letter of Ethics Committee of the First Affiliated Hospital, Collage of Medicine, Zhejiang University, and the approval number is 2017-623. After which, mononuclear cells were separated by density centrifugation with Ficoll-Paque (Thermo Fisher Scientific, USA). The interlayer cells were collected and cultured in a cell culture flask with Chang medium (S-Evans Biosciences, Hangzhou, China) in an incubator set at 37 °C under an atmosphere with 5% CO_2_, with medium changes performed every 2–3 days. Subsequently, the cells were subcultured with 0.25% trypsin-EDTA (Thermo Fisher Scientific) until reaching 80–90% confluence and were used in experiments before passage 8. STR identification of MenSCs has been performed before use which is listed in Additional file 7.

The lentiviral luciferase expression vector Ubi-MCS-firefly_Luciferase-IRES-Puromycin was purchased from Genechem (Shanghai, China), and the viral titer was calculated to be 2 × 10^8^/ml transducing units. MenSCs were used at passage 1/2 at a multiplicity of infection of 50 for 12 h before being selected with 2 μg/ml puromycin (Beyotime, Haimen, China) for 24 h.

The immortalized mouse lung type II epithelial cell line murine lung epithelia-12 (MLE-12) was purchased from the American Type Culture Collection (Manassas, VA, USA) and cultured in Dulbecco’s modified Eagle’s medium/Nutrient Mixture F-12 (DMEM/F12, Thermo Fisher Scientific) supplemented with 10% fetal bovine serum (FBS; Thermo Fisher Scientific) and 2 mM l-glutamine (Thermo Fisher Scientific).

Primary mouse lung fibroblasts (MLFs) were isolated from the lungs of 6-week-old male C57BL/6J mice. Briefly, the mice were anesthetized with 1% pentobarbital sodium (100 mg/ml), after which the lung tissues were washed, minced, and digested with type 1 collagenase (Sigma, San Francisco, USA) for 45 min at 37 °C before being cultured in DMEM (Corning, California, USA) supplemented with 10% FBS. MLFs were purified from macrophages by passage and used in experiments before passage 5.

### Surface makers and differentiation of MenSCs

Approximately 5 × 10^5^ MenSCs were collected and suspended with stain buffer (BD, Biosciences, San Jose, CA) twice before being incubated with antibodies targeting surface makers, including phycoerythrin (PE)-conjugated CD29, CD34, CD45, CD73, CD90, CD105, and CD117 and human leukocyte antigen-DR (HLA-DR) (BD, Biosciences, San Jose, USA) in the dark at 4 °C for 15–30 min; the specific information is listed in Table S1. Then, the stained cells were washed twice with stain buffer, and corresponding isotype antibodies were used as negative controls. All cells were analyzed with a flow cytometer (FC500MCL, Beckman Coulter, Pasadena).

For the detection of differentiation potential, human mesenchymal stem cell osteogenic differentiation medium, chondrogenic differentiation medium, and adipogenic differentiation medium A and B (Cyagen Biosciences, USA) were used according to the manufacturer’s instructions. MenSCs were used in passage 5. Briefly, for adipogenic differentiation, MenSCs were cultured for 2–4 weeks in human mesenchymal stem cell adipogenic differentiation complete medium A containing 10% FBS, 1% penicillin-streptomycin, 1% glutamine, 0.2% insulin, 0.01% rosiglitazone, 0.01% dexamethasone, and 0.01% 3-isobutyl-1-methylxanthine and in human mesenchymal stem cell adipogenic differentiation complete medium B containing 10% FBS, 1% penicillin-streptomycin, 1% glutamine, and 0.2% insulin. The cells were washed with phosphate-buffered saline (PBS; pH 7.4), fixed with 4% formaldehyde at room temperature for 10 min, and incubated with Oil Red O solution for 30 min at room temperature to label eutral lipids. For osteogenic differentiation, MenSCs were cultured for 2–4 weeks in human mesenchymal stem cell osteogenic differentiation complete medium containing 10% FBS, 1% penicillin-streptomycin, 1% glutamine, 0.2% ascorbate, 1% β-glycerophosphate, and 0.01% dexamethasone. The cells were washed with PBS, fixed with 4% formaldehyde at room temperature for 10 min, and incubated with Alizarin Red at room temperature for 30 min to label calcium. For chondrogenic differentiation, 2.5 × 10^5^ MenSCs were transferred to a 15-ml polypropylene tube and centrifuged. Then, the pelleted cells were cultured at 37 °C under an atmosphere with 5% CO_2_ in 0.5 ml of human mesenchymal stem cell chondrogenic differentiation complete medium containing 0.01% dexamethasone, 0.3% ascorbate, 1% insulin-transferrin-sodium supplement, 0.1% sodium pyruvate, 0.1% proline, and 1% transforming growth factor-β3. The medium was replaced with fresh medium every 2–3 days for 4 weeks. Pelleted cells were fixed with 4% formaldehyde at room temperature for 10 min, embedded in paraffin, and then cut into 4-mm-thick sections. Subsequently, the sections were stained with Alcian blue solution for 30 min at room temperature to label sulfated cartilage glycosaminoglycans (GAGs).

### BLM-induced pulmonary fibrosis and MenSC transplantation

To induce pulmonary fibrosis, C57BL/6J mice were anesthetized with 1% pentobarbital sodium (100 mg/ml). Then, 3 U/kg of BLM dissolved in 50 μl phosphate-buffered saline (PBS) was intratracheally administered to the mice, after which 5 × 10^5^ MenSCs in 500 μl of PBS were injected into the tail vein of the mice 2 and 7 days after BLM administration, with this group referred to as the MenSC group. Mice injected with an equal volume of PBS were referred to as the BLM group, and normal C57BL/6J mice served as a blank control. After obtaining MenSCs that stably expressed the luciferase gene, they were injected into mice via the tail vein. And a live imaging system was used to observe the migration of MenSCs.

### Histopathological analysis, fibrotic examination, hydroxyproline (HYP) content detection, and immunohistochemistry

C57BL/6J mice were anesthetized with 1% pentobarbital sodium on day 21. The body weights of mice were measured, and blood samples were collected and stored in a refrigerator at 4 °C overnight. Then, each sample was centrifuged at 2000×*g* and 4 °C for 10 min to obtain serum, which was then aliquoted and frozen at − 80 °C for subsequent experiments. For each mouse, the left lung was weighed and dried at 55 °C overnight, and the dry/wet ratio was reported as the dry to wet weight. The upper right lung was fixed with 4% paraformaldehyde, embedded in paraffin, and then stained with hematoxylin and eosin (H&E) and Masson. At least five random fields of view of each group were imaged with an Olympus IX-83-FV3000-OSR instrument (Olympus Corporation, Japan).

The HYP content was measured to assess the collagen deposition in the lung tissues using an HYP detection kit (Nanjing Jiancheng Bioengineering Institute, Nanjing, China) according to the manufacturer’s instructions. Briefly, lung tissue homogenates were hydrolyzed in 6 M hydrochloric acid for 5 h at 95 °C, after which chloramine-T added and the pH was adjusted. Subsequently, after the addition of a color developer, the samples were incubated for 15 min at 60 °C, and the absorbance at 550 nm was measured using a Microplate Reader (Bio-Rad, USA). Hydroxyproline levels were determined by plotting a standard curve, and the results are presented as micrograms per milligram in lung tissues.

Immunohistochemistry was performed as described in our previous study [22]. Briefly, 5-μm paraffin sections were dewaxed with xylene and rehydrated in an alcohol gradient. Then, the samples were incubated for 20 min with 10 mM sodium citrate buffer (pH 6.0) for antigen retrieval with simultaneous heating before being incubated for 10 min with 3% hydrogen peroxide to block endogenous peroxidases. Next, sections were incubated at 4 °C overnight with the following primary antibodies listed in Table S1 and incubated with corresponding HRP-conjugated secondary antibodies for 1 h at room temperature.

### Bronchoalveolar lavage fluid collection, cell counting, and enzyme-linked immunosorbent assays

Briefly, each trachea was cannulated and lavaged twice with 0.5 ml of sterile PBS at room temperature. Then, after the bronchoalveolar lavage fluid (BALF) was centrifuged at 500×*g* for 10 min at 4 °C, the supernatants were stored at − 20 °C until testing, and the cell pellets were resuspended in 200 μl of PBS for cell counting and cell smears. Blood samples were collected and centrifuged at 4 °C and 2000×*g* 10 min to obtain serum samples.

For the BALF and serum samples from three groups of mice, the levels of interleukin-10 (IL-10), interleukin-1β (IL-1β), interleukin-6 (IL-6), and transforming growth factor-β1 (TGF-β1) were quantified using enzyme-linked immunosorbent assay kits (R&D systems, USA) according to the manufacturer’s instructions, with the results presented as picogrammes per milliliter.

### Coculture experiments: MenSC/MLE-12 cells and MenSCs/MLFs

MenSCs and MLE-12 cells were seeded onto transwell inserts at a 1:1 ratio, with MenSCs and MLE-12 cells seeded into the upper and lower chambers, respectively. MLE-12 cells were stimulated with 5 ng/ml of BLM for 24 h in DMEM/F12 medium and then washed with PBS twice before being cocultured without MenSCs. The three assay groups were as follows: the control group, MLE-12 cells; the BLM group, MLE-12 cells treated with BLM; and the MenSC group, MLE-12 cells treated with BLM and cocultured with MenSCs.

Similarly, MenSCs and MLFs were seeded onto transwell inserts at a 1:1 ratio (0.4-μm pore size; Corning), with MenSCs and MLFs were seeded in the upper and lower chambers, respectively. MLFs were stimulated with 3 ng/ml of TGF-β1 (Peprotech, Rocky Hill, NJ, USA) for 48 h in MEM medium without FBS and then washed with PBS twice before being cocultured with MenSCs for 72 h. The three assay groups were as follows: the control group, MLFs; the TGF-β1 group, MLFs treated with TGF-β1; and the MenSC group, MLFs treated with TGF-β1 and cocultured with MenSCs.

### Proliferation and clonogenic assays

For proliferation and clonogenic assays, 2 × 10^4^ MLF/MLE-12 cells and MenSCs were seeded into the lower and upper transwell chambers of 24-well plates, respectively. A cell counting kit-8 (CCK8; Dojindo Molecular technologies, Rockville, MD) was used to assess cell viability according to the manufacturer’s instructions. Briefly, CCK8 reagent was added to the medium and incubated for 2 h in the dark. Subsequently, the absorbance of each sample at 450 nm was measured using a microplate reader, and the experiment was repeated in triplicate.

MLE-12 cells were seeded in 6-well plates at a density of 1500 cells per well in three different groups and cultured for 7 days. Then, the cells were fixed with 4% paraformaldehyde and stained with a crystal violet solution. The number of colonies was calculated with an Olympus IX-83-FV3000-OSR, and the experiment was repeated in triplicate.

### Apoptosis and cell cycle analysis

For apoptosis and cell cycle analysis, 2 × 10^5^ MLE-12 cells and MenSCs were seeded into the lower and upper transwell chambers of 6-well plates, respectively. An Annexin V/PI detection kit (BD Biosciences, CA, USA) was used to detect MLE-12 apoptosis, and a Cell Cycle and Apoptosis analysis kit (Beyotime Biotechnology, Haimen, China) was used to detect the MLE-12 cell cycle. Both experiments were performed according to the manufacturer’s instructions using a FC500 flow cytometer, and the data were analyzed using FlowJo software (Tree Star, OR, USA).

To determine the number of lung epithelial cells in lung tissues, flow cytometry was performed. The lung tissues were cut into small pieces and digested with type 1 collagenase for 1 h at 37 °C. Then a single-cell suspension was obtained by grinding the digested tissue through a 100-μm filter (BD Biosciences, CA, USA) before being centrifuged at 300×*g* for 10 min at 4 °C, and red blood cells were lysed twice. Subsequently, the cells were resuspended with stain buffer (BD Biosciences) and incubated with antibodies listed in Additional file 5, Table S1 for 30 min at 4 °C. Subsequently, the cells were washed twice with stain buffer before being analyzed with a multicolor flow cytometer (BD Biosciences). The data were analyzed using FlowJo software.

### Immunofluorescence staining

MLF/MLE-12 cells were washed with PBS 3 times and fixed with 4% paraformaldehyde for 30 min at room temperature. Subsequently, the cells were permeabilized with enhanced immunostaining permeabilization buffer for 20 min before being blocked with goat serum (Beyotime, haimen, China) for 1 h at room temperature. Then, the MLFs were incubated with anti-α-SMA, anti-fibronectin, and anti-collagen1antibodies (listed in Additional file 5, Table S1), while MLE-12 cells were incubated with antibodies against E-cadherin and N-cadherin (listed in Additional file 5, Table S1) at 4 °C overnight, which was followed by incubations with Alexa Fluo-488-conjugated goat anti-mouse and Alexa Fluo-635-conjucated goat anti-rabbit antibodies for 1 h. After being washed with PBST 3 times, the samples were covered with ProLong™ Gold antifade mountant with DAPI and coverslips, and images were taken with an Olympus IX-83-FV3000-OSR microscope.

Immunofluorescence analysis was also performed to determine whether MenSCs differentiate into the injury site cells. Briefly, lung tissues from mice in the three groups were embedded in Tissue-Tek O.C.T. compound (Sakura Finetek, Torrance, CA), after which 5-μm-thick sections were incubated with a human surfactant protein D antibody at 4 °C overnight and then incubated with an Alexa Fluo-635-conjugated goat anti-mouse IgG antibody. Next, the sections were covered with ProLong™ gold antifade mountant with DAPI and coverslips. Finally, images were taken with an Olympus IX-83-FV3000-OSR microscope.

### Scratch assay and transwell invasion assay

A culture insert (IBIDI) was used to observe healing in scratched areas. For this assay, 7 × 10^4^ MLE-12 cells were seeded into the two sides of a culture insert. The culture insert was then removed with forceps after 24 h and washed with PBS twice to remove floating cells, after which fresh medium supplemented with 5 ng/ml of BLM was added to the wells. The MLE-12 cells were then cocultured with MenSCs after BLM stimulation for 24 h, and images were taken at 0 and 48 h to calculate the scratch area, with the experiment repeated in triplicate. We use ImageJ software to calculate the scratch area at different time points.

For transwell invasion assays, 8-μm-pore-sized upper chambers were pretreated with matrigel (BD Biosciences, USA) for 1 h at 37 °C. Then, 2 × 10^4^ MLE-12 cells in 100 μl of serum-free medium treated with BLM were added to the upper chambers after seeding 2 × 10^4^ MenSCs in the lower chamber. After coculturing for 48 h, the cells in the upper chambers were fixed with 4% paraformaldehyde and stained with a crystal violet solution. The number of stained cells was calculated using an Olympus IX-83-FV3000-OSR microscope, and the experiment was repeated in triplicate.

### Quantitative real-time PCR and western blotting

Total RNA was extracted from cultured MLFs using a total RNA kit (Qiagen) according to the manufacturer’s instructions. Reverse transcription was performed using a PrimeScript™ RT reagent kit (Takara), and real-time PCR was performed with a SYBR Premix Ex TaqTM kit (Takara) on an ABI 7500 Fast Real-Time PCR Systems instrument (Thermo Fisher Scientific). The primers used for quantitative real-time PCR are listed in Additional file 6, Table S2. The data were analyzed using the 2^−△△Ct^ method to calculate relative RNA levels, which were normalized to GAPDH for each sample.

Total proteins were extracted from lung tissues using cell lysis buffer for western blot and immunoprecipitation assays supplemented with a protease and phosphatase inhibitor cocktail. Tissue lysate was centrifuged at 12,000 rpm for 10 min at 4 °C, and the BCA method was used to determine protein concentration. Then, 8–12% SDS-PAGE (5% stacking gels at 80 V, 8–12% resolving gels at 120 V) was used to separate proteins, which were then transferred to PVDF membranes (200 mA, 1 h). After being blocked with 5% skim milk for 2 h at room temperature, the membranes were incubated overnight at 4 °C with primary antibodies listed in Additional file 5, Table S1. After being washed with TBST, the membranes were incubated with secondary antibodies (goat anti-rabbit IgG (HCL)-HRP conjugate (1:3000; Bio-Rad) and goat anti-mouse IgG (HCL)-HRP conjugate (1:3000; Bio-Rad)) for 1 h at room temperature and then detected using a Tanon-4500 gel imaging system.

### RNA-Seq and mouse cytokine array

Five lung tissue samples were selected from mice in each of three groups (control, BLM, and MenSC groups). Then, total RNA was extracted using a total RNA kit (Qiagen) according to the manufacturer’s instructions, and genomic DNA was removed using DNase I (TaKara). Then, the RNA quality was determined using a 2100 Bioanalyzer instrument (Agilent) and quantified using an ND-2000 instrument (NanoDrop Technologies). Only high-quality RNA samples were used to construct sequencing libraries. After quantification using a TBS380 instrument, paired-end RNA-Seq of the library was performed using an Illumina HiSeq 4000 platform. The raw RNA-Seq data produced in this study has been submitted to the NCBI Gene Expression Omnibus (GEO; https://www.ncbi.nlm.nih.gov/geo) under accession number PRJNA514227.

A RayBio® Mouse Cytokine Antibody Array (QAM-CYT-4-8) was used to detect the levels of 200 cytokines secreted by MLE-12 cells (*n* = 3/group). Cell culture supernatants were harvested after culturing for 48 h in the MLE-12, MLE-12 + BLM, and MLE-12 + BLM + MenSC groups. The expression of cytokines was determined by measuring the fluorescent intensities following the manufacturer’s instructions.

### Statistics

Statistical analysis was performed using GraphPad Prism 6 (GraphPad, San Diego, CA). Student’s *t* test and one-way analysis of variance was used to test for significant differences between two or more groups, respectively. The data are presented as the means±SD from at least three independent experiments, and *p* values < 0.05 were considered significant.

## Results

### Identification of MenSCs

**Fig. 1:**
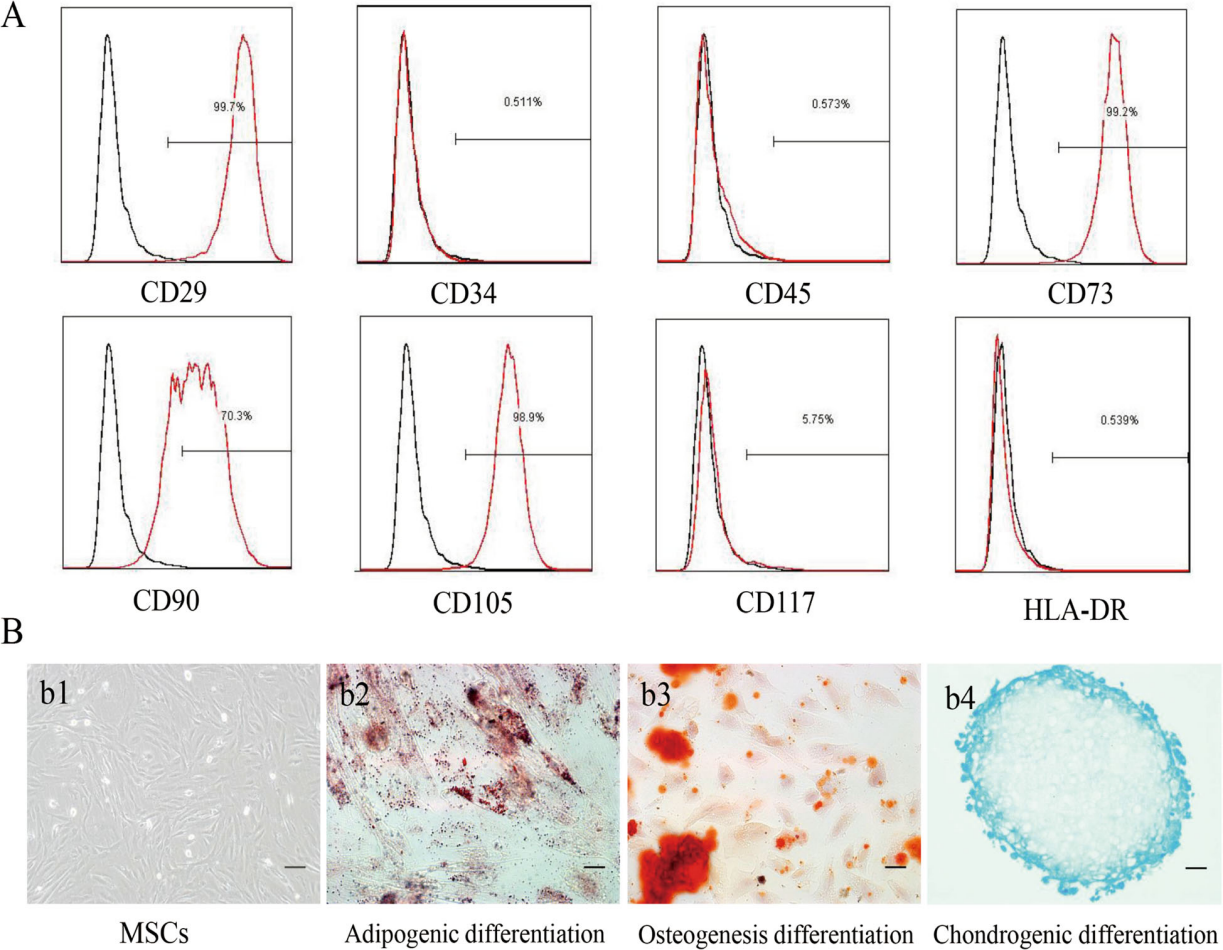
Characterization of MenSCs. **a** Surface makers of MenSCs were analyzed by a flow cytometer. The black line represents isotype controls, while the red line represents the levels of the surface markers. **b** Representative images of MenSCs (b1) and differential potential of MenSCs, Oil Red O staining of intracellular neutral lipid vacuoles (b2), Alizarin red staining of calcium deposition (b3), Alcian blue staining of sulfated cartilage GAGs (b4). Scale bar: 100 μm

Flow cytometry was performed to identify the immunophenotype of MenSCs, with cells exhibiting positive expression of CD29, CD73, CD90, and CD105 (Fig. 1a) and negative expression of CD34, CD45, CD117, and HLA-DR (Fig. 1a). MenSCs showed a spindle and fibroblast-like shape (Fig. 1b) and could be successfully differentiated into adipogenic, osteogenic, and chondrogenic cells using specific medium (Fig. 1b).

### MenSCs reduce BLM-induced apoptosis and EMT of MLE-12 cells

**Fig. 2:**
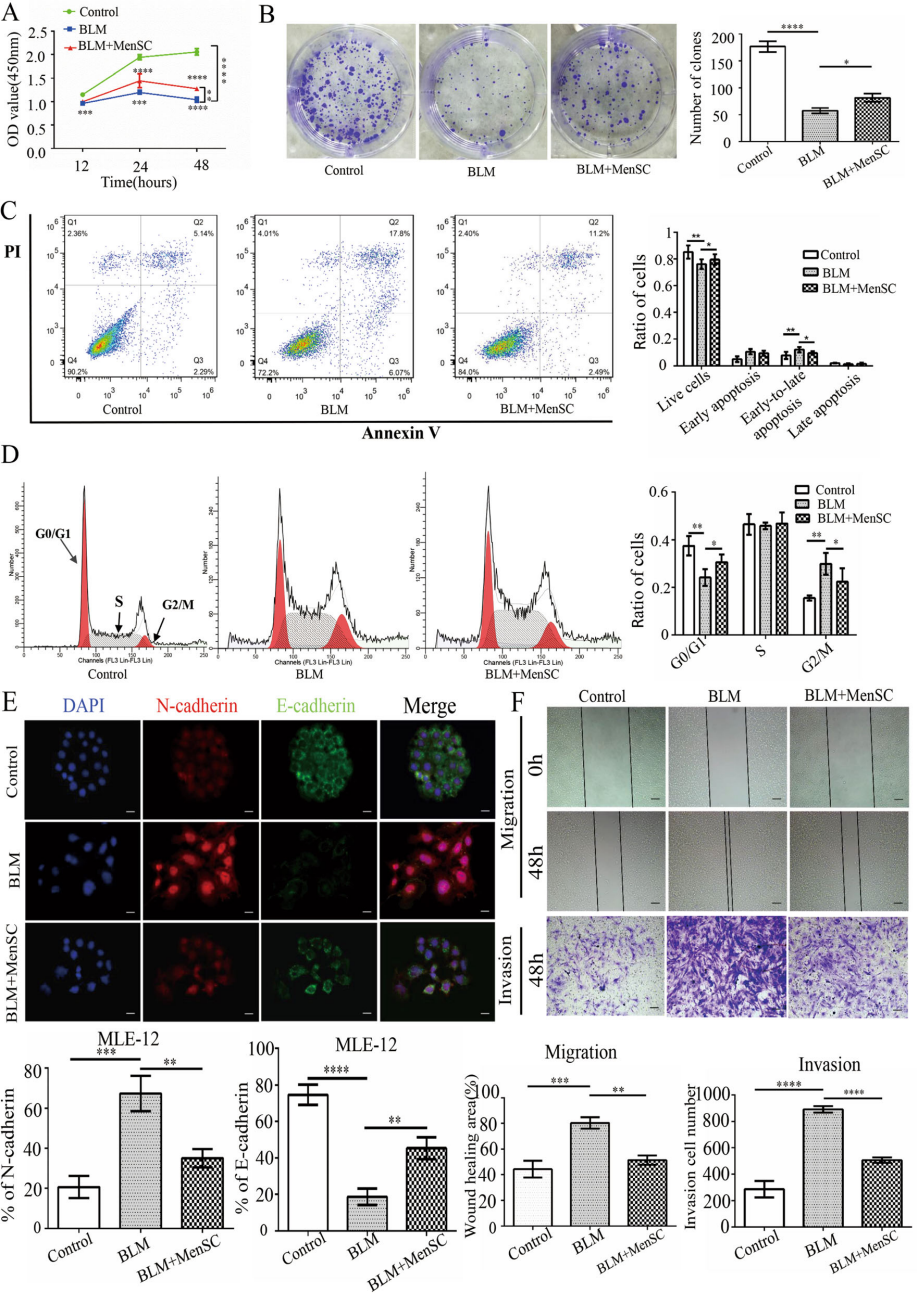
MenSCs reduced BLM-induced apoptosis and EMT of MLE-12 cells. **a** The anti-apoptosis effect of MenSCs on BLM-treated MLE-12 cells examined by CCK8 assay after coculture for 48 h (*n* = 4/time point). **b** Clonal formation ability of BLM-treated MLE-12 cells examined by the clonogenic assay (*n* = 3). Cell apoptosis (**c**) and cell cycle (**d**) of BLM-treated MLE-12 cells was performed by flow cytometry (*n* = 3). **e** The levels of E-cadherin and N-cadherin protein of BLM-treated MLE-12 cells were measured by IF after cocultured with MenSCs (*n* = 3). Scale bar: 20 μm. **f** Migration and invasion of BLM-treated/untreated MLE-12 cells were examined by the wound healing assay and transwell assay after coculture with MenSCs (*n* = 5). Scale bar: 50 μm. Error bars indicate standard deviation of three biological replicates undergoing independent differentiations. *p < 0.05 and **p < 0.01, ***p < 0.001, ****p < 0.0001 by one-way analysis of variance

We measured the biological effect of MenSCs on the proliferation of MLE-12 cells, with CCK8 assay results showing that MenSCs significantly reduced BLM-induced MLE-12 cell injury (Fig. 2a) and promoted their clonogenic potential compared with the BLM group (Fig. 2b). Additionally, flow cytometry assay results confirmed that cocultured with MenSCs, the rate of MLE-12 cell apoptosis was significantly reduced (Fig. 2c), and MenSCs significantly attenuated BLM-induced cell cycle arrest in the G2/M phase (Fig. 2d). These results indicate that MenSCs have an anti-apoptosis effect on the proliferation of MLE-12 cells treated with BLM.

To assess whether MenSCs have an impact on BLM-induced epithelial-mesenchymal transition (EMT), we investigated the expression of EMT-related proteins. Immunofluorescence assay results showed that E-cadherin levels significantly increased after MLE-12 cells were cocultured with MenSCs, while that of N-cadherin significantly decreased (Fig. 2e). Furthermore, wounding healing and invasion assay results showed that the migration and invasion ability of BLM-treated MLE-12 cells was significantly inhibited by MenSCs (Fig. 2f). These results suggested that MenSCs can inhibit BLM-induced EMT in MLE-12 cells.

### MenSCs inhibit the TGF-β1-induced proliferation and differentiation of MLFs

**Fig. 3:**
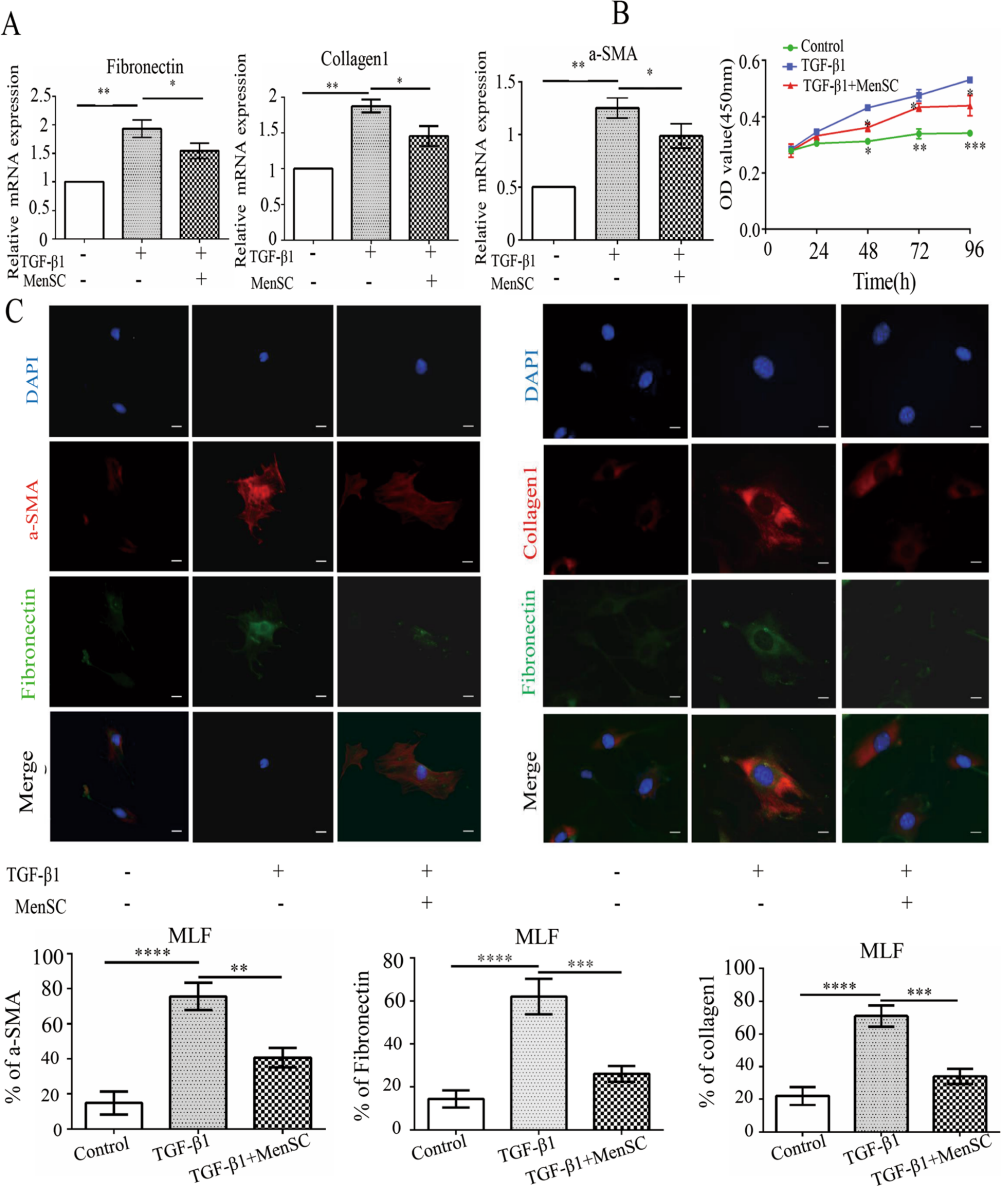
MenSCs attenuate TGF-β1-induced differentiation and proliferation of mouse lung fibroblasts (MLFs). **a** The mRNA levels of fibronectin, collagen1, and a-SMA stimulated after stimulation with TGF-β1 and coculture with MenSCs (*n* = 3). **b** CCK8 assay in MLF cells after stimulation with TGF-β1 and coculture with MenSCs (*n* = 4/time point). **c** Immunofluorescence assay in MLFs cells after stimulation with TGF-β1 and coculture with MenSCs (*n* = 3). Scale bar: 20 μm. Error bars indicate standard deviation of three biological replicates undergoing independent experiments. *p < 0.05, **p < 0.01, ***p < 0.001 by one-way analysis of variance

Extensive extracellular matrix (ECM) deposition is also a pathological feature of pulmonary fibrosis, and fibroblast proliferation and differentiation cause massive ECM formation [24]. To access the impact of MenSCs on TGF-β1-treated MLFs, we measured collagen1, α-SMA, and fibronectin levels in MLFs after being cocultured with MenSCs for 72 h using real-time PCR and immunofluorescence assays. The results showed that collagen 1, α-SMA, and fibronectin expression in MLFs was decreased after being cocultured with MenSCs (Fig. 3a), and immunofluorescence data also confirmed this result (Fig. 3c). Additionally, CCK8 assays were performed to investigate the impact of MenSCs on the proliferation of MLFs, with the results showing that the number of MLFs was approximately the same in the three groups at 12 h. However, after 24 h, the proliferation of MLFs was significantly inhibited by MenSCs (Fig. 3b), indicating that MenSCs have a suppressive impact on the differentiation and proliferation of MLFs.

### MenSCs migrate to the injured lung and reduce lung epithelial cell apoptosis in vivo

**Fig. 4:**
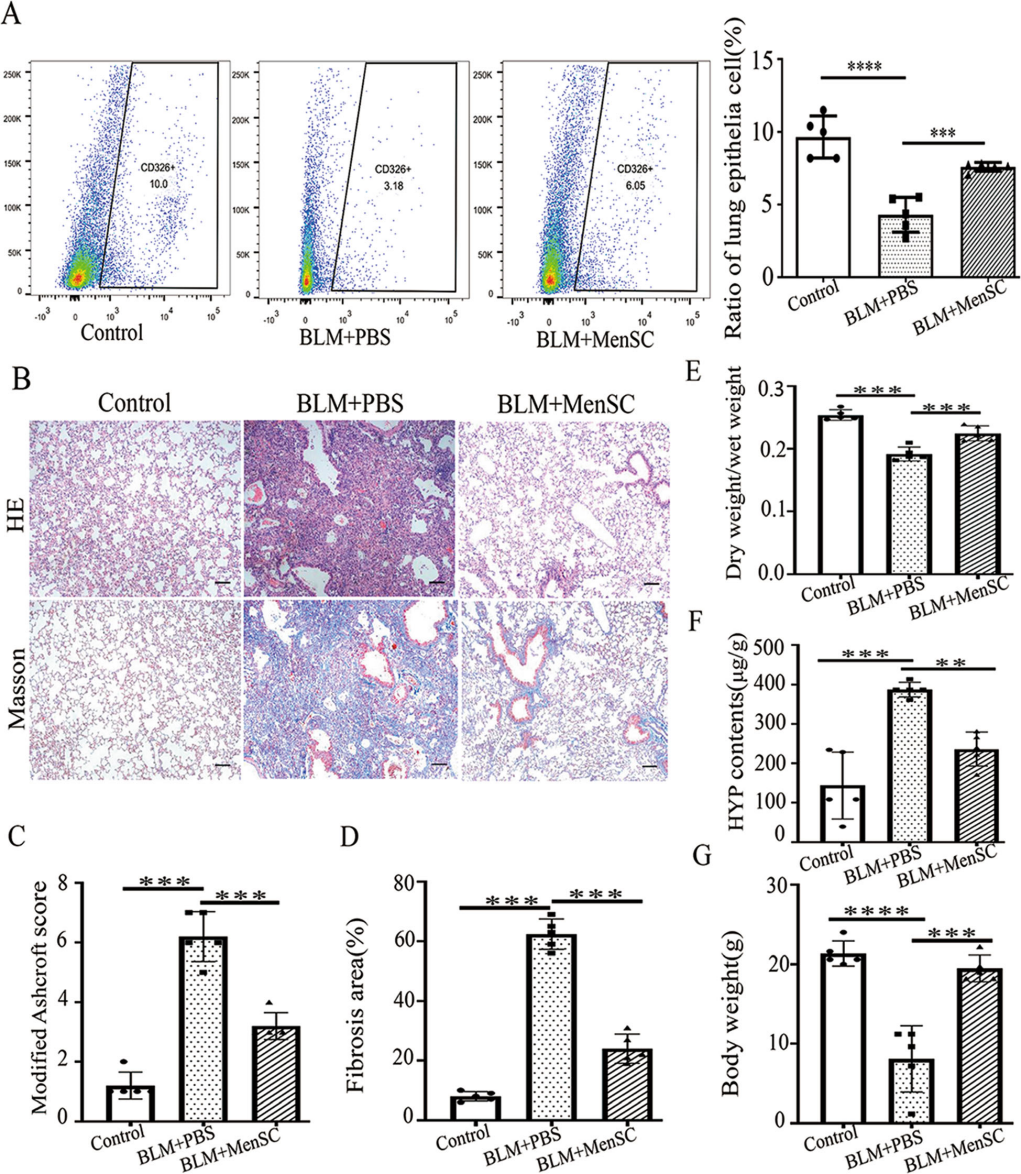
MenSC transplantation reduced epithelia cell apoptosis in vivo and repaired the lung structure following fibrosis. **a** Lung epithelia cell ratio in three different groups (*n* = 5). **b** H&E staining and Masson staining of lung sections (*n* = 5). Scale bar: 50 μm. Severity of pulmonary fibrosis evaluated by modified Ashcroft score **(c)**, fibrosis area **(d)**, and HYP contents **(f)**. **e** Dry/wet weight ratio analysis (*n* = 5). **g** Body weight of mice in three different groups (*n* = 5). Error bars indicate standard deviation of five biological replicates undergoing independent experiments. *p < 0.05, **p < 0.01, ***p < 0.001 by one way analysis of variance

To investigate the effects of MenSCs on BLM-induced pulmonary fibrosis, MenSCs expressing luciferase were transplanted into mice through tail vein injection twice a week after BLM administration. Live imaging results showed that the BLM-treated group recruited more MenSCs that migrated into the injured lung compared with control group (listed in additional file 1, Figure S1A), while the observation of low human specific-surfactant protein D (SPD) expression revealed that few MenSCs differentiated into lung epithelial cells (listed in additional file 1, Figure S1B). Furthermore, to assess whether MenSCs had a protective effect on lung epithelial cell as observed in vitro, multicolor flow cytometry was performed. According to the results, the BLM group displayed fewer epithelial cells compared with the control group, while this apoptosis was reduced after MenSC administration (Fig. 4a). These results indicated that MenSCs migrated to the injured lung and exhibited an anti-apoptosis effect in vivo.

### MenSCs mitigate the symptoms of pulmonary fibrosis

H&E and Masson staining were performed to observed lung structure and assess collagen deposition in the lung interstitium of the three different groups. The H&E and Masson staining results showed that mice from the BLM-induced group exhibited serious damage to the structure of the lung alveoli, large amounts of inflammatory cell infiltration, and massive amounts of collagen especially around the bronchi and vessels compared with the control group (Fig. 4b). In contrast, the alveolar structure, number of inflammatory cells, and collagen deposition were decreased compared with the BLM group after MenSC transplantation (Fig. 4b). In addition, the fibrosis area and modified Ashcroft scores corresponded to the section observation (Fig. 4c, d). MenSC transplantation also increased the dry/wet radio of lung tissues (Fig. 4e). HYP detection can aid in assessing the degree of fibrosis at a semi-quantitative level according to the collagen contents. A significant increase in collagen deposition was detected in the BLM group compared with the control group, while MenSC transplantation reduced the deposition of collagen deposition compared with the BLM group (Fig. 4f), which was consistent with the Masson staining results. The body weights of mice in the BLM group showed a significant reduction compared with the control group 21 days after BLM administration, and a recovery trend was observed after MenSC transplantation (Fig. 4g). These results demonstrate that the administration of MenSCs improved the symptoms of pulmonary fibrosis.

### MenSCs attenuate inflammation in pulmonary fibrosis mouse

**Fig. 5:**
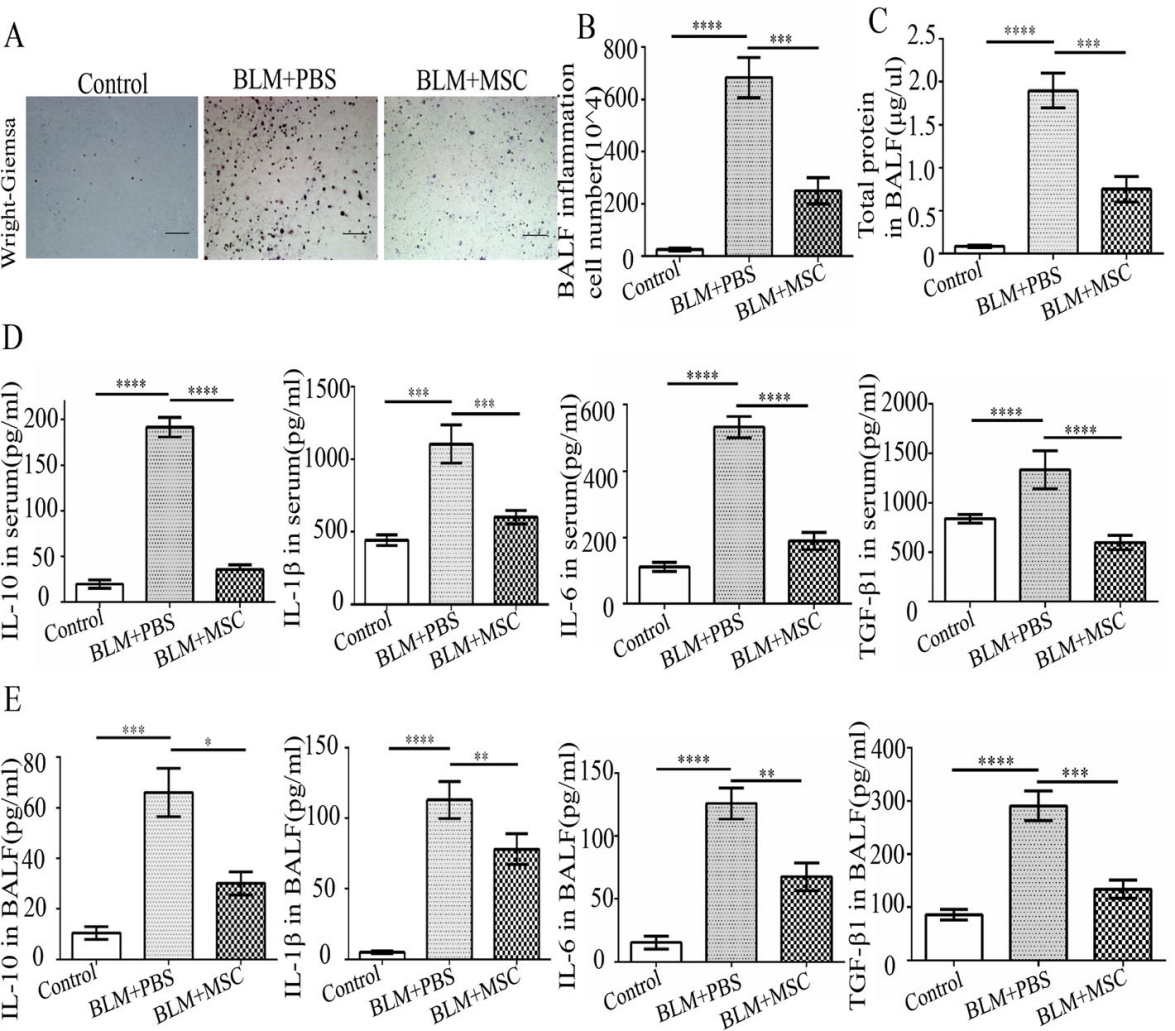
MenSC transplantation mitigated lung inflammation. **a**, **b** Inflammatory cells in BALF were observed by cell smears and counts (*n* = 5). Scale bar: 50 μm. **c** The total proteins in BALF. **d**, **e** Levels of IL-10, IL-1β, IL-6, and TGF-β1 in serum and BALF in three different groups. Error bars indicate standard deviation of five biological replicates undergoing independent experiments. *p < 0.05 and **p < 0.01, ***p < 0.001, ****p < 0.0001 by one-way analysis of variance

The BALF and serum inflammatory cytokines levels reflect the level of inflammation. Therefore, the levels of inflammatory cytokines were evaluated to assess whether MenSCs could mitigate lung inflammation. According to the cell smear results, the number of inflammatory cells in the BLM group was significantly increased compared with the control group but decreased after MenSC administration (Fig. 5a). Total BALF protein was increased in the BLM group but decreased in the MenSC group compared with the control group (Fig. 5b, c). In addition, the level of BALF-associated inflammatory cytokines showed the same trend. After MenSC administration, the levels of IL-10, IL-1β, IL-6, and TGF-β1 were decreased (Fig. 5d). In addition, the serum concentrations of IL-10, IL-1β, IL-6, and TGF-β1 exhibited the same trend as observed in BALF (Fig. 5e). These results indicate that MenSCs have a potential effect on immunoregulation for reducing lung inflammation.

### Different cytokines expression in three different groups

**Fig. 6:**
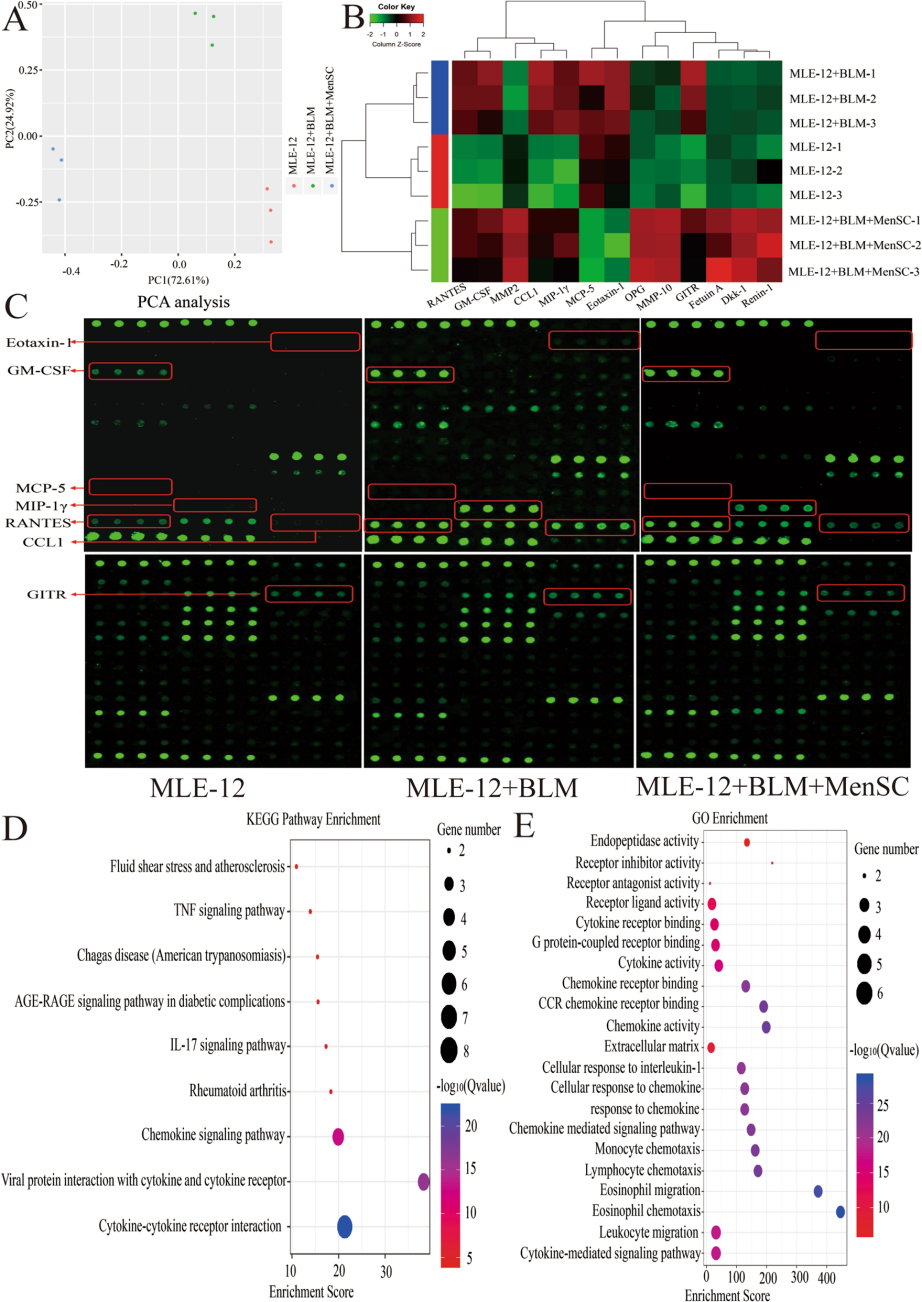
Cytokine detection in BLM-treated MLE-12 cells after MenSC coculture. **a** PCA analysis of three different groups (*n* = 3). **b** Differential cytokine expression shown as a heat map. Red, green, and black colors represent upregulated, downregulated, and unchanged expression, respectively (*n* = 3). **c** Cytokines of significantly expressed in three different groups (RANTES, GM-CSF, MIP-1γ, MCP-5, Eotaxin, CCL1, and GITR) are highlighted with red boxes and arrows (*n* = 3). **d**, **e** Significantly different results from GO (**c**) and KEGG pathway (**d**) analysis results. Three biological replicates undergoing independent experiments were performed

To assess the suppressive effect of MenSCs on BLM-induced MLE-12 cell apoptosis, we used an antibody array (Ray Biotech; www.raybiotech.com) to examine cytokine levels in the supernatants of the MLE-12, MLE-12 + BLM, and MLE-12 + BLM + MenSC groups. Among the 200 cytokines evaluated (listed in Additional file 2, Figure S2), some showed low or even undetectable expression (data not shown). PCA analysis showed few differences within the same groups, while different groups could be distinguished by a large separation (Fig. 6a). As shown in Fig. 6b, c, the levels of several cytokines (i.e., RANTES, GM-CSF, MIP-1γ, MCP-5, Eotaxin, CCL1 and GITR) were significantly decreased in the MLE-12 + BLM + MenSC group compared to the MLE-12 + BLM group. GO and KEGG pathway enrichment results showed that inflammation factors, chemotaxis, and cytokine-cytokine receptor interaction pathways may be the most important pathways in this process (Fig. 6d, e).

### MenSCs relieve BLM-induced pulmonary fibrosis through anti-fibrosis and anti-apoptosis effects

**Fig. 7:**
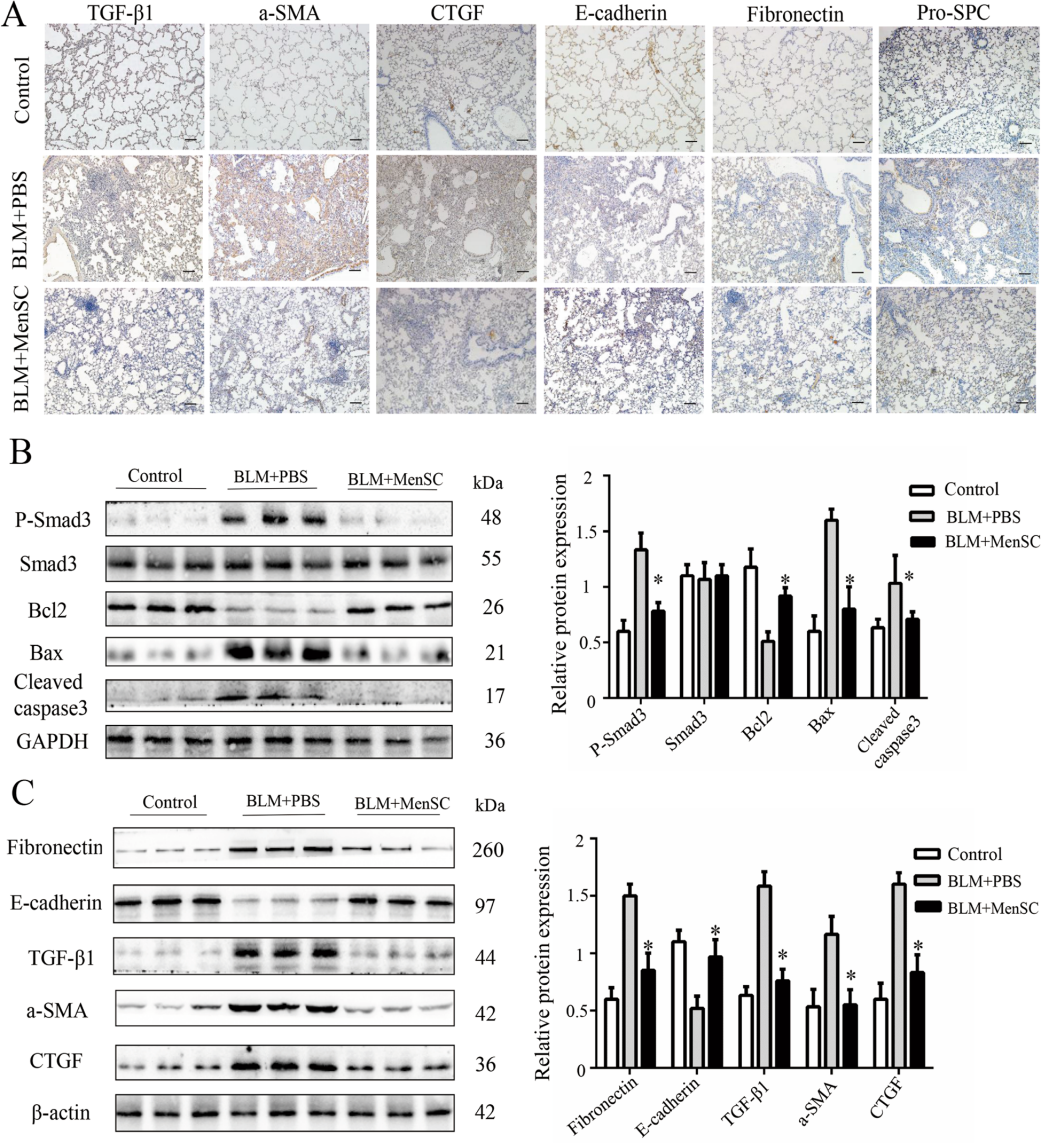
MenSC transplantation promotes lung repair and reduced the expression of fibrosis-related proteins. **a** Representative immunohistochemistry images for TGF-β1, fibronectin, E-cadherin, CTGF, a-SMA, and Pro-SPC (*n* = 3). Scale bar: 100 μm. **b** Western blot analysis protein levels of P-Smad3, Smad3, Cleaved caspase3, Bcl2, Bax, and GAPDH in the lung tissues of three different groups (*n* = 3). **c** Fibronectin, E-cadherin, TGF-β1, a-SMA, CTGF, and β-actin in the lung tissues of three different groups (*n* = 3). Error bars indicate standard deviation of three biological replicates undergoing independent experiments. *p < 0.05 by one-way analysis of variance

To elucidate how the identified differentially expressed genes are associated with BLM-induced pulmonary fibrosis, total RNA was extracted from the lung tissues of mice in the control, BLM, and MenSC groups and used for RNA-Seq analysis on an Illumina HiSeq platform. A total of 2503 genes were identified as being significantly differentially expressed between the control and BLM groups, while 252 significantly differentially expressed genes were identified between the BLM and MenSC groups (listed in Additional file 3, Figure S3A). Among these genes, 955 genes were upregulated while 1548 genes were downregulated between the control and BLM groups, and 188 genes were upregulated while 64 were downregulated between the BLM and MenSC groups (listed in Additional file 3, Figure S3B). GO and KEGG enrichment analyses were performed to further analyze the pathways for the 210 common significant differentially expressed genes among the three groups. We observed that muscle contraction was involved in most processes and that the expression of genes involved in the Apelin signaling pathway, focal adhesion, and NF-kappa B signaling, which are associated with the development of fibrosis, was significantly altered (listed in Additional file 3, Figure S3C and D). Additionally, we also observed that the expression of α-SMA, CTGF, and cdh1, which are involved in the apelin signaling pathway, was associated with renal fibrosis (listed in Additional file 4, Figure S4), leading us to speculate that this pathway may play an important role in the process of pulmonary fibrosis. We measured the in situ and total protein expression levels in the mouse lung tissues after MenSC transplantation through immunohistochemical (IHC) and western blot analyses. As expected, the IHC results showed that after MenSC transplantation, the expression of TGF-β1, CTGF, and α-SMA proteins significantly decreased, whereas that of E-cadherin was increased compared to that observed in the BLM group (Fig. 7a). The western blot assays showed similar results to those obtained from IHC (Fig. 7c), and we also measured the expression of upstream proteins, with the results showing that MenSC transplantation reduced the expression of Smad3 phosphorylation (Fig. 7b), although the phosphorylation of Smad2 and Smad4 showed no significant difference (data not shown). Moreover, with respect to the expression of the apoptosis-related proteins Bcl2/Bax/Cleaved caspase-3, the results presented in Fig. 7b show that the MenSC treatment resulted in a significant increase in Bcl2 levels and decreased BAX and cleaved caspase-3 levels compared with the BLM group. According to these results, we speculated that MenSCs play an important role in the anti-apoptosis effect in a BLM-induced pulmonary fibrosis.

## Discussion

Pulmonary fibrosis is a fetal and progressive disease with an increasing global mortality rate. Although both nintedanib and pirfenidone are approved by the FDA for the treatment of pulmonary fibrosis, these drugs only slow the progression of this disease [10, 25]. Currently, the transplantation of MSCs is increasingly conducted in preclinical and clinical studies due to their potential in tissue regeneration and immunoregulation [26]. Previous studies reported that MSC transplantation may promote lung repair and regulate the process of inflammation to reduce fibrosis [9, 27], with the systemic transplantation of MSCs firstly demonstrating fibrotic-ameliorating effects in BLM-induced mice [28]. Several clinical studies also have shown that transplantation of MSC from placenta, bone marrow, umbilical cord, adipose tissue, and other sources to patients with moderate or severe IPF is safe in a short term, which can improve the quality of life and respiratory parameters of patients. It is speculated that MSC may be a safe and effective treatment for IPF [29–33]. In this study, MenSCs were shown to have multiple effects in anti-fibrotic therapy, namely, MenSCs migrated to the injured lung significantly reduced collagen deposition in lung interstitium and the systemic inflammatory response, and repaired the damage of the lung structure in a pulmonary fibrosis mouse model.

The excessive accumulation of ECM proteins caused by the imbalance between collagen synthesis and degradation is the primarily pathological features of pulmonary fibrosis, which results in poor gas exchange and eventually leads to respiratory failure [34]. Activated fibroblasts are one of the primary source of myofibroblasts, which secrete extracellular matrix (ECM). In addition, epithelia cells transdifferentiate into fibroblast-like cells and fibrocytes via EMT and are also a major source of myofibroblasts [35]. Dmytro’s studies have shown that lung epithelia cells exposed to BLM enter oxidative stress and undergo apoptosis or EMT [36]. Our group has demonstrated that MenSCs could attenuate EMT in hepatocellular carcinoma and inhibit the activation and proliferation of hepatic stellate cells to attenuate liver fibrosis [21, 23]. Similarly, we found that MenSCs significantly inhibit BLM-induced EMT via downregulating the expression of N-cadherin and upregulating the expression of E-cadherin in MLE-12 cells. In addition, we also observed that MenSCs exhibited a suppressing effect on the proliferation and differentiation of primary lung fibroblasts in vitro. These results indicated that MenSCs have great potential in reducing the source of myofibroblasts in vitro.

Several studies suggested that MSCs can express alveolar epithelial-specific markers after induction by a specific medium, including surfactant protein C and thyroid transcription factor 1 [37, 38]. At the same time, it has been found in vivo that GFP-MSCs also migrate to damaged lungs and express lung epithelial cell markers [39, 40]. However, others have shown that MSCs do not differentiate into epithelial cells, but can promote the repair and regeneration of alveolar epithelium through paracrine effects [41]. Although our group has demonstrated that MenSCs can differentiate into lung epithelia-like cells in vitro, the present study showed that few MenSCs differentiated into alveolar epithelial cells, although MenSCs migrated to damaged sites and reduced epithelial cell apoptosis in vivo. We speculate that it is not necessary for the differentiation of MenSCs to promote lung repair.

During the process of pulmonary fibrosis, epithelia/endothelia cells were damaged in the earliest stage, and then released inflammatory cytokines to initiate a cascade reaction [35]. Mounting evidence has shown that the apoptosis of type II alveolar epithelial cells leads to persistent and excessive migration and activation of fibroblast in the lung interstitium, leading to subsequent generation and accumulates myofibroblasts, which is closely associated with the progression of pulmonary fibrosis. It is likely that loss of the anti-fibrotic characteristics of intact epithelial cells and the increasing secretion of profibrotic factors from injured epithelial cells causes fibrosis [42]. In the present study, we also found that MenSCs can alleviate BLM-induced apoptosis of MLE-12 cells in vitro. A similar study also demonstrated the ability of MSCs to protect alveolar epithelia cells (AECs) against apoptosis [39], in contrast to a study showing that MSCs differentiate into AECs through a paracrine mechanism [43].

Despite the strong relationship between AEC apoptosis and pulmonary fibrosis, the underlying mechanism still requires further research. Some reports have shown that IL-6 played a cytoprotective role in type 2 pneumocytes of BLM-induced lung injury [44], and hepatocyte growth factor (HGF) promoted epithelia repair in fibrotic lung disease [8]. Our group has demonstrated that MenSCs secrete high levels of IL-6 and HGF [45], so we hypothesized that MenSCs may function through different cytoprotective factors to inhibit the apoptosis of AECs. Subsequently, we detected a significantly reduced expression of cytokines (including RANTES, Eotaxin, GM-CSF, MIP-1γ, MCP-5, CCL1, GITR) using antibody arrays in MLE-12 cells and MenSC coculture experiments, and bioinformatics analysis results showed that cytokine-cytokine receptor interactions may be involved in this process.

Chemokines have various biological functions in different diseases. RANTES (also known as CCL5) and Eotaxin (also known as CCL11) are important chemokines of Eos, which can directly mobilize Eos to migrate into the airway lumen, leading to the enhancement of the inflammatory response [46]. Compared with normal people, patients with pulmonary fibrosis expressed higher RANTES and Eotaxin in bronchoalveolar lavage fluid, and these chemokines help recruit eosinophils to the injured lung [47]. In addition, a significant increase of the number of leukocytes was accompanied with the elevated expression by RANTES and T cell secretion of profibrotic factors in bleomycin-treated mice [48]. Moreover, in the AECs of radiotherapy patients, RANTES expression was significantly upregulated with EMT, while inhibiting RANTES expression also blocked its promotion effect on the EMT of AECs [49]. GM-CSF is massively produced and activated at the site of inflamed tissue, and it has a therapeutic effect in different inflammatory diseases, including cardiac inflammation during aortic aneurysm formation and interstitial lung disease [50]. Other studies have shown that excessive inhalation of smoke can cause an increased expression of GM-CSF in the lungs, and GM-CSF can stimulate the proliferation and activation of alveolar macrophages, which can lead to emphysema or secondary polycythemia and increase mortality [51]. MIP-1γ (also known as CCL9) is a murine ligand of the CCR1/CD191 receptor present on T cells, monocytes, and some immature CD34 ^+^ myeloid-derived suppressor cells. It can drive macrophage recruitment and angiogenesis, as well as T-cell- and B-cell-dependent PD-L1 excretion, and CCR1 is associated with tumorigenesis and the pathology of inflammatory lung diseases [15, 52]. We observed that RANTES, Eotaxin, and GM-CSF were highly expressed in MLE-12 cells after BLM treatment, while the expression in the MenSC group was significantly reduced. AEC apoptosis triggers inflammation and recruits a large number of inflammatory cells to reach the lungs and secrete different cytokines to promote the development of fibrosis [35]. Many reports have shown that MSCs have great potential in providing immunomodulatory effects and attenuating the inflammatory response and fibrosis, but the underlying mechanism is still not clear. However, the therapeutic effect of MSCs is related to the number of transplantated cells and the timing of transplantation. Compared with late-stage transplantation (data not shown), we found that early transplantation of MenSCs is more efficient because late-stage transplantation is more likely to cause vascular embolism. At the same time, we speculate that early transplantation of MenSCs may suppress inflammation in the initial stage and delay the progression of pulmonary fibrosis.

Similarly, Murine CCL12 (also known as MCP-5) is an analog of human CCL2, and CCL2 and its receptor CCR2 are one of the most widely studied chemokines and receptors in pulmonary fibrosis. The expression of CCL12 in lung tissue of pulmonary fibrosis mice is significantly increased, and CCR2^−/−^mice do not develop pulmonary fibrosis [53, 54]. However, it is still controversial whether CCL2^−/−^ mice can be protected from pulmonary fibrosis. Recent studies have shown that one target of CCL2 may be CCR2 ^+^ CD4 ^+^ T cells. These T cells have similar functions to those of regulatory T cells and have been found to have certain anti-fibrotic effects in animal models [55]. In addition, CCL12 knockout mice were found to have a compensatory increase in the expression of CCL2 and CCL7 in lung tissue and bronchoalveolar lavage fluid, and CCL12 knockout did not protect mice from the formation of pulmonary fibrosis. In contrast, in mice in which the CCL12 gene in alveolar epithelial cells has been conditionally knocked out, the expression of CCL2 and CCL7 in lung tissue and bronchoalveolar lavage fluid was not different from that in normal control mice, confirming that the conditional knockout of the CCL12 gene in alveolar epithelial cells can protect mice from developing pulmonary fibrosis [56]. Consistent with our research, we found that in MLE-12 cells treated with bleomycin, MCP-5 was significantly elevated than that in normal MLE-12 cells, but the expression of MCP-5 was significantly reduced after coculture with MenSCs. Previous studies have shown that CCL2 is highly expressed in AECs of IPF patients [18]. It is indicated that MCP-5 may be a potential target for the future therapy of MenSCs.

Currently, COVID-19 is sweeping the world like a plague. It is a SARS-like virus that spread rapidly in Wuhan and even spread for a period of time throughout China, and there are no specific and effective drugs or vaccines [57]. Some patients are infected with severe pneumonia and died of respiratory failure [58]. The symptoms are similar to those of patients with pulmonary fibrosis in the late period [59, 60]. MSCs have been shown to exhibit powerful immune regulation function, and transplantation of MSCs is considered to be one of the most feasible, safe, and effective ways to treat severe pneumonia patients. Leng et al. found that intravenous transplantation of MSCs significantly improved or cured the functional outcomes of those patients with severe pneumonia, without any adverse effects [61]. MenSC therapy has achieved excellent results in the treatment of H7N9, greatly reducing the mortality caused by H7N9. At the same time, after a 5-year return visit, MenSCs did not show any side effects on the patient [62]. According to the research on the therapeutic effect of MenSCs in H7N9 patients and pulmonary fibrosis mice, we speculate that MenSC transplantation may become one of the available treatment options for COVID-19 treatment. However, additional studies are needed to further verify the feasibility of this treatment.

## Conclusions

In conclusion, the probable underlying mechanism of MenSCs is that the inhibition of epithelial cell apoptosis and inflammatory response involves a reduction in the secretion of inflammatory factors or chemokines (including RANTES, Eotaxin, GMS-CF, MCP-5, CCL1, and GITR), which prevents myofibroblast activity and collagen deposition, hindering the development of fibrosis. In addition, we demonstrated that MenSCs attenuated BLM-induced pulmonary fibrosis in mice, and these results indicate that MenSCs could provide a therapeutic strategy for treating patients with pulmonary fibrosis in clinical applications.

## Supplementary information

**Additional file 1.**

**Additional file 2.**

**Additional file 3.**

**Additional file 4.**

**Additional file 5.**

**Additional file 6.**

**Additional file 7.**

## Data Availability

RNA-Seq data in this study have been submitted to the NCBI Gene Expression Omnibus (GEO; https://www.ncbi.nlm.nih.gov/geo) under accession number PRJNA514227.

## Abbreviations

BLM: Bleomycin
CCK-8: Cell Counting Kit-8
CCL1: Chemokine1
CCL5: Chemokine5
CCR2: Chemokine receptor 2
COVID-19: Coronavirus Disease 2019
CTGF: Connective tissue growth factor
ECM: Extracellular matrix
EDTA: Ethylenediaminetetraacetic acid
ELISA: Enzyme-linked immunosorbent assays
EMT: Epithelial-mesenchymal transition
FBS: Fetal bovine serum
GITR: Glucocorticoid-induced tumor necrosis factor receptor
GM-CSF: Granulocyte-macrophage colony stimulating factor
IFN-γ: Interferon gamma
IL-6: Interleukin 6
MenSCs: Menstrual blood-derived stem cells
MCP-5: Monocyte chemoattractant protein 5
MIP-1γ: Macrophage Inflammatory Protein*-1γ*
MSC: Mesenchymal stem cell
OD: Optical value
PBS: Phosphate-buffered saline
RANTES: Regulated upon activation normal T cell expressed and secreted factor
RT-PCR: Reverse transcription-polymerase chain reaction
TBST: Tris-buffered saline with tween
TGF-β: Transform growth factor β

## References

[1] El Agha E, Kramann R, Schneider RK, Li X, Seeger W, Humphreys BD (2017). Mesenchymal stem cells in fibrotic disease. Cell Stem Cell.

[2] Sime PJ, Xing Z, Graham FL, Csaky KG, Gauldie J (1997). Adenovector-mediated gene transfer of active transforming growth factor-β1__induces prolonged severe fibrosis in rat lung. J Clin Invest.

[3] Ian Y. R. Adamson, H. D, Bowden. Origin of Ciliated Alveolar Epithelial Cells in__Bleomycin-Induced Lung Injury. Am J Pathol. 1977:569–80. PMC2032137 68683

[4] Naikawadi RP, Disayabutr S, Mallavia B, Donne ML, Green G, La JL (2016). Telomere dysfunction in alveolar epithelial cells causes lung remodeling and fibrosis. JCI Insight.

[5] Caplan AI, Dennis JE (2006). Mesenchymal stem cells as trophic mediators. J Cell Biochem.

[6] Lan YW, Choo KB, Chen CM, Hung TH, Chen YB, Hsieh CH (2015). Hypoxia-preconditioned mesenchymal stem cells attenuate bleomycin-induced pulmonary fibrosis. Stem Cell Res Ther.

[7] Lan YW, Theng SM, Huang TT, Choo KB, Chen CM, Kuo HP (2017). Oncostatin M-preconditioned mesenchymal stem cells alleviate bleomycin-induced pulmonary fibrosis through paracrine effects of the hepatocyte growth factor. Stem Cells Transl Med.

[8] Cahill EF, Kennelly H, Carty F, Mahon BP, English K. Hepatocyte growth factor is required for mesenchymal stromal cell protection against bleomycin-induced pulmonary fibrosis. Stem Cells Transl Med. 2016;5(10):1307–18. doi: 10.5966/sctm.2015-0337. Epub 2016/07/09. PubMed PMID: 27388243; PubMed Central PMCID: PMCPMC5031177. 10.5966/sctm.2015-0337 PMC5031177 27388243

[9] Chen S, Cui G, Peng C, Lavin MF, Sun X, Zhang E, et al. Transplantation of adipose-derived mesenchymal stem cells attenuates pulmonary fibrosis of silicosis via anti-inflammatory and anti-apoptosis effects in rats. Stem Cell Res Ther. 2018;9(1):110. doi: 10.1186/s13287-018-0846-9. Epub 2018/04/21. PubMed PMID: 29673394; PubMed Central PMCID: PMCPMC5909257. 10.1186/s13287-018-0846-9 PMC5909257 29673394

[10] Reddy M, Fonseca L, Gowda S, Chougule B, Hari A, Totey S. Human adipose-derived mesenchymal stem cells attenuate early stage of bleomycin induced pulmonary fibrosis: comparison with pirfenidone. Int J Stem Cells. 2016;9(2):192–206. doi: 10.15283/ijsc16041. Epub 2016/11/23. PubMed PMID: 27871152; PubMed Central PMCID: PMCPMC5155715. 10.15283/ijsc16041 PMC5155715 27871152

[11] Zhang C, Yin X, Zhang J, Ao Q, Gu Y, Liu Y. Clinical observation of umbilical cord mesenchymal stem cell treatment of severe idiopathic pulmonary fibrosis: a case report. Exp Ther Med. 2017;13(5):1922–6. doi: 10.3892/etm.2017.4222. Epub 2017/06/02. PubMed PMID: 28565787; PubMed Central PMCID: PMCPMC5443299. 10.3892/etm.2017.4222 PMC5443299 28565787

[12] Allickson J, Xiang C. Human adult stem cells from menstrual blood and endometrial tissue. J Zhejiang Univ Sci B. 2012;13(5):419–20. Epub 2012/05/05. doi: 10.1631/jzus. B1200062. PubMed PMID: 22556182; PubMed Central PMCID: PMCPMC3348235. 10.1631/jzus.B1200062 PMC3348235 22556182

[13] Meng X, Ichim TE, Zhong J, Rogers A, Yin Z, Jackson J, et al. Endometrial regenerative cells: a novel stem cell population. J Transl Med. 2007;5:57. doi: 10.1186/1479-5876-5-57. Epub 2007/11/17. PubMed PMID: 18005405; PubMed Central PMCID: PMCPMC2212625. 10.1186/1479-5876-5-57 PMC2212625 18005405

[14] Khoury M, Alcayaga-Miranda F, Illanes SE, Figueroa FE. The promising potential of menstrual stem cells for antenatal diagnosis and cell therapy. Front Immunol. 2014;5:205. doi: 10.3389/fimmu.2014.00205. Epub 2014/06/07. PubMed PMID: 24904569; PubMed Central PMCID: PMCPMC4032935. 10.3389/fimmu.2014.00205 PMC4032935 24904569

[15] Kortlever RM, Sodir NM, Wilson CH, Burkhart DL, Pellegrinet L, Brown Swigart L, et al. Myc cooperates with Ras by programming inflammation and immune suppression. Cell. 2017;171(6):1301–15 e14. doi: 10.1016/j.cell.2017.11.013. Epub 2017/12/02. PubMed PMID: 29195074; PubMed Central PMCID: PMCPMC5720393. 10.1016/j.cell.2017.11.013 PMC5720393 29195074

[16] Nikoo S, Ebtekar M, Jeddi-Tehrani M, Shervin A, Bozorgmehr M, Vafaei S (2014). Menstrual blood-derived stromal stem cells from women with and without endometriosis reveal different phenotypic and functional characteristics. Mol Hum Reprod.

[17] Jiang Z, Hu X, Yu H, Xu Y, Wang L, Chen H, et al. Human endometrial stem cells confer enhanced myocardial salvage and regeneration by paracrine mechanisms. J Cell Mol Med. 2013;17(10):1247–60. doi: 10.1111/jcmm.12100. Epub 2013/07/11. PubMed PMID: 23837896; PubMed Central PMCID: PMCPMC3843975. 10.1111/jcmm.12100 PMC3843975 23837896

[18] Mercer PF, Johns RH, Scotton CJ, Krupiczojc MA, Konigshoff M, Howell DC, et al. Pulmonary epithelium is a prominent source of proteinase-activated receptor-1-inducible CCL2 in pulmonary fibrosis. Am J Respir Crit Care Med. 2009;179(5):414–25. doi: 10.1164/rccm.200712-1827OC. Epub 2008/12/09. PubMed PMID: 19060230; PubMed Central PMCID: PMCPMC2648910. 10.1164/rccm.200712-1827OC PMC2648910 19060230

[19] Wu X, Luo Y, Chen J, Pan R, Xiang B, Du X, et al. Transplantation of human menstrual blood progenitor cells improves hyperglycemia by promoting endogenous progenitor differentiation in type 1 diabetic mice. Stem Cells Dev. 2014;23(11):1245–57. doi: 10.1089/scd.2013.0390. Epub 2014/02/07. PubMed PMID: 24499421; PubMed Central PMCID: PMCPMC4027987. 10.1089/scd.2013.0390 PMC4027987 24499421

[20] Mou XZ, Lin J, Chen JY, Li YF, Wu XX, Xiang BY, et al. Menstrual blood-derived mesenchymal stem cells differentiate into functional hepatocyte-like cells. J Zhejiang Univ Sci B. 2013;14(11):961–72. doi: 10.1631/jzus. Epub 2013/11/06. B1300081. PubMed PMID: 24190442; PubMed Central PMCID: PMCPMC3829645. 10.1631/jzus.B1300081 PMC3829645 24190442

[21] Chen L, Zhang C, Chen L, Wang X, Xiang B, Wu X, et al. Human menstrual blood-derived stem cells ameliorate liver fibrosis in mice by targeting hepatic stellate cells via paracrine mediators. Stem Cells Transl Med. 2017;6(1):272–84. doi: 10.5966/sctm.2015-0265. Epub 2017/02/09. PubMed PMID: 28170193; PubMed Central PMCID: PMCPMC5442725. 10.5966/sctm.2015-0265 PMC5442725 28170193

[22] Xiang B, Chen L, Wang X, Zhao Y, Wang Y, Xiang C. Transplantation of menstrual blood-derived mesenchymal stem cells promotes the repair of LPS-induced acute lung injury. Int J Mol Sci. 2017;18(4). doi: 10.3390/ijms18040689. Epub 2017/03/28. PubMed PMID: 28346367; PubMed Central PMCID: PMCPMC5412275. 10.3390/ijms18040689 PMC5412275 28346367

[23] Wu Y, Chen X, Zhao Y, Wang Y, Li Y, Xiang C. Genome-wide DNA methylation and hydroxymethylation analysis reveal human menstrual blood-derived stem cells inhibit hepatocellular carcinoma growth through oncogenic pathway suppression via regulating 5-hmC in enhancer elements. Stem Cell Res Ther. 2019;10(1):151. doi: 10.1186/s13287-019-1243-8. Epub 2019/06/04. PubMed PMID: 31151404; PubMed Central PMCID: PMCPMC6544940. 10.1186/s13287-019-1243-8 PMC6544940 31151404

[24] Herrera J, Henke CA, Bitterman PB. Extracellular matrix as a driver of progressive fibrosis. J Clin Invest. 2018;128(1):45–53. doi: 10.1172/JCI93557. PubMed PMID: 29293088; PubMed Central PMCID: PMCPMC5749528. 10.1172/JCI93557 PMC5749528 29293088

[25] Graney BA, Lee JS. Impact of novel antifibrotic therapy on patient outcomes in idiopathic pulmonary fibrosis: patient selection and perspectives. Patient Relat Outcome Meas. 2018;9:321–8. doi: 10.2147/PROM.S144425. Epub 2018/10/06. PubMed PMID: 30288134; PubMed Central PMCID: PMCPMC6163010. 10.2147/PROM.S144425 PMC6163010 30288134

[26] Weiss ARR, Dahlke MH. Immunomodulation by mesenchymal stem cells (MSCs): mechanisms of action of living, apoptotic, and dead MSCs. Front Immunol. 2019;10:1191. doi: 10.3389/fimmu.2019.01191. Epub 2019/06/20. PubMed PMID: 31214172; PubMed Central PMCID: PMCPMC6557979. 10.3389/fimmu.2019.01191 PMC6557979 31214172

[27] Liu D, Kong F, Yuan Y, Seth P, Xu W, Wang H (2018). Decorin-modified umbilical cord mesenchymal stem cells (MSCs) attenuate radiation-induced lung injuries via regulating inflammation, fibrotic factors, and immune responses. Int J Radiat Oncol Biol Phys.

[28] Ortiz LA, Gambelli F, McBride C, Gaupp D, Baddoo M, Kaminski N (2003). Mesenchymal stem cell engraftment in lung is enhanced in response to bleomycin exposure and ameliorates its fibrotic effects. PNAS..

[29] Chambers DC, Enever D, Ilic N, Sparks L, Whitelaw K, Ayres J (2014). A phase 1b study of placenta-derived mesenchymal stromal cells in patients with idiopathic pulmonary fibrosis. Respirology..

[30] Marilyn K. Glassberg M, Julia Minkiewicz P, Rebecca L. Toonkel M, Emmanuelle S. Simonet M, Gustavo A. Rubio M, Darcy DiFede R, BSN; , et al. Allogeneic human mesenchymal stem cells in patients with idiopathic pulmonary fibrosis via intravenous delivery (AETHER)_ A Phase I Safety Clinical Trial. chest. 2017:151(5):971–81. doi: 10.1016/j.chest.2016.10.061. 10.1016/j.chest.2016.10.061 PMC6026255 27890713

[31] Chunyu Zhang, Xiaoguang Yin, Jinghan Zhang, Qiang Ao, Gu Y, Liu Y. Clinical observation of umbilical cord mesenchymal stem cell treatment of severe idiopathic pulmonary fibrosis_ a case report. Experimental and therapeutic medicine. 2017;13:1922–6. 10.3892/etm.2017.4222. 10.3892/etm.2017.4222 PMC5443299 28565787

[32] Averyanov A, Koroleva I, Konoplyannikov M, Revkova V, Lesnyak V, Kalsin V (2019). First-in-human high-cumulative-dose stem cell therapy in idiopathic pulmonary fibrosis with rapid lung function decline. Stem Cells Transl Med.

[33] Argyris Tzouvelekis, Vassilis Paspaliaris, George Koliakos, Paschalis Ntolios, Evangelos Bouros, Anastasia Oikonomou, et al. A prospective, non-randomized, no__placebo-controlled, phase Ib clinical trial to study__the safety of the adipose derived stromal__cells-stromal vascular fraction in idiopathic pulmonary fibrosis. Journal of clinical medicine.11:171:2–13. 10.1186/1479-5876-11-171 PMC3722100 23855653

[34] Michael J. Evans, Linda J. Cabral, BS RJ, Stephens, Gustave Freeman. Renewal of alveolar epithelium in the Rat__following exposure to NO2. Am J Pathol 1973:175–190. PMC1903972 4566990

[35] Wynn TA. Integrating mechanisms of pulmonary fibrosis. J Exp Med. 2011;208(7):1339–50. doi: 10.1084/jem.20110551. Epub 2011/07/06. PubMed PMID: 21727191; PubMed Central PMCID: PMCPMC3136685. 10.1084/jem.20110551 PMC3136685 21727191

[36] Petukhov D, Richter-Dayan M, Fridlender Z, Breuer R, Wallach-Dayan SB. Increased regeneration following stress-induced lung injury in bleomycin-treated chimeric mice with CD44 knockout mesenchymal cells. Cells. 2019;8(10). doi: 10.3390/cells8101211. Epub 2019/10/09. PubMed PMID: 31591327; PubMed Central PMCID: PMCPMC6829612. 10.3390/cells8101211 PMC6829612 31591327

[37] Sueblinvong V, Loi R, Eisenhauer PL, Bernstein IM, Suratt BT, Spees JL (2008). Derivation of lung epithelium from human cord blood-derived mesenchymal stem cells. Am J Respir Crit Care Med.

[38] Ma N, Gai H, Mei J, Ding FB, Bao CR, Nguyen DM (2011). Bone marrow mesenchymal stem cells can differentiate into type II alveolar epithelial cellsin vitro. Cell Biol Int.

[39] Rojas M, Xu J, Woods CR, Mora AL, Spears W, Roman J (2005). Bone marrow-derived mesenchymal stem cells in repair of the injured lung. Am J Respir Cell Mol Biol.

[40] Lang L, Dong L, Zhang J, Gao F, Hui J, Yan J (2019). Mesenchymal stem cells with downregulated Hippo signaling attenuate lung injury in mice with lipopolysaccharide-induced-acute respiratory distress syndrome. Int J Mol Med.

[41] Wei L, Zhang J, Yang ZL, You H (2017). Extracellular superoxide dismutase increased the therapeutic potential of human mesenchymal stromal cells in radiation pulmonary fibrosis. Cytotherapy..

[42] Kim KK, Dotson MR, Agarwal M, Yang J, Bradley PB, Subbotina N (2018). Efferocytosis of apoptotic alveolar epithelial cells is sufficient to initiate lung fibrosis. Cell Death Dis.

[43] Li L, Liang D, Qianzhang J. Mesenchymal stem cells with downregulated Hippo signaling attenuate lung injury in mice with lipopolysaccharide-induced acute respiratory distress syndrome. Iternational J Mol Med. 2018:1241–52. 10.3892/ijmm.2018.4047. 10.3892/ijmm.2018.4047 PMC6365074 30628652

[44] Kobayashi T, Tanaka K, Fujita T, Umezawa H, Amano H, Yoshioka K (2015). Bidirectional role of IL-6 signal in pathogenesis of lung fibrosis. Respir Res.

[45] Chen L, Xiang B, Wang X, Xiang C (2017). Exosomes derived from human menstrual blood-derived stem cells alleviate fulminant hepatic failure. Stem Cell Res Ther.

[46] Eun Kyung Ryu, Tae-Hyun Kim, Eun Jeong Jang. Wogonin, a plant flavone from Scutellariae radix, attenuated ovalbumininduced airway inflammation in mouse model of asthma via the suppression of IL4/STAT6 signaling. J Clin Biochem Nutr. 2015:105–12. doi: 10.3164/jcbn.15 45. 10.3164/jcbn.15-45 PMC4566018 26388667

[47] Emad A, Emad Y (2008). Relationship between eosinophilia and levels of chemokines (CCL5 and CCL11) and IL-5 in bronchoalveolar lavage fluid of patients with mustard gas-induced pulmonary fibrosis. J Clin Immunol.

[48] Russo RC, Alessandri AL, Garcia CC, Cordeiro BF, Pinho V, Cassali GD (2011). Therapeutic effects of evasin-1, a chemokine binding protein, in bleomycin-induced pulmonary fibrosis. Am J Respir Cell Mol Biol.

[49] Zhong Y, Lin Z, Lu J, Lin X, Xu W, Wang N (2019). CCL2-CCL5/CCR4 contributed to radiation-induced epithelial-mesenchymal transition of HPAEpiC cells via the ERK signaling pathways. Am J Transl Res.

[50] Becher B, Tugues S, Greter M (2016). GM-CSF: from growth factor to central mediator of tissue inflammation. Immunity..

[51] Suzuki T, McCarthy C, Carey BC, Borchers M, Beck D, Wikenheiser-Brokamp KA (2020). Increased pulmonary GM-CSF causes alveolar macrophage accumulation. Mechanistic implications for desquamative interstitial pneumonitis. Am J Respir Cell Mol Biol.

[52] Kitamura T, Qian BZ, Soong D, Cassetta L, Noy R, Sugano G (2015). CCL2-induced chemokine cascade promotes breast cancer metastasis by enhancing retention of metastasis-associated macrophages. J Exp Med..

[53] Baran CP, Opalek JM, McMaken S, Newland CA, O'Brien JM, Hunter MG (2007). Important roles for macrophage colony-stimulating factor, CC chemokine ligand 2, and mononuclear phagocytes in the pathogenesis of pulmonary fibrosis. Am J Respir Crit Care Med..

[54] Larson-Casey JL, Vaid M, Gu L, He C, Cai GQ, Ding Q (2019). Increased flux through the mevalonate pathway mediates fibrotic repair without injury. J Clin Invest.

[55] Milger K, Yu Y, Brudy E, Irmler M, Skapenko A, Mayinger M (2017). Pulmonary CCR2(+)CD4(+) T cells are immune regulatory and attenuate lung fibrosis development. Thorax..

[56] Yang J, Agarwal M, Ling S, Seagal Teitz-Tennenbaum LR, Zemans, et al. (2020). Diverse injury pathways induce alveolar epithelial cell CCL2_12, which promotes lung fibrosis. Am J Respir Cell Mol Biol.

[57] Cohen J, Normile D (2020). New SARS-like virus in China triggers alarm. Science (New York, NY).

[58] Zhou F, Yu T, Du R, Fan G, Liu Y, Liu Z (2020). Clinical course and risk factors for mortality of adult inpatients with COVID-19 in Wuhan, China: a retrospective cohort study. Lancet.

[59] Ahn DG, Shin HJ, Kim MH, Lee S, Kim HS, Myoung J (2020). Current status of epidemiology, diagnosis, therapeutics, and vaccines for novel coronavirus disease 2019 (COVID-19). J Microbiol Biotechnol.

[60] Rothan HA, Byrareddy SN (2020). The epidemiology and pathogenesis of coronavirus disease (COVID-19) outbreak. J Autoimmun.

[61] Leng Z, Zhu R, Hou W, Feng Y, Yang Y, Han Q (2020). Transplantation of ACE2(−) mesenchymal stem cells improves the outcome of patients with COVID-19 pneumonia. Aging Dis.

[62] Chen J, Hu C, Chen L, Tang L, Zhu Y, Xu X, et al. Clinical study of mesenchymal stem cell treating acute respiratory distress syndrome induced by epidemic Influenza A (H7N9) infection, a hint for COVID-19 treatment. Engineering (Beijing). 2020. doi: 10.1016/j.eng.2020.02.006. Epub 2020/04/16. PubMed PMID: 32292627; PubMed Central PMCID: PMCPMC7102606. 10.1016/j.eng.2020.02.006 PMC7102606 32292627

