# Supplementary material for: Human menstrual blood-derived stem cells mitigate bleomycin-induced pulmonary fibrosis through anti-apoptosis and anti-inflammatory effects
Source: Stem Cell Res Ther. 2020 Nov 11;11:477. doi: 10.1186/s13287-020-01926-x (PMC7656201; doi:10.1186/s13287-020-01926-x)
Supplement: Supplementary file 1 — Additional file 1. [file 13287_2020_1926_MOESM1_ESM.pdf]

Additional file 1

Supplemental Figure 1

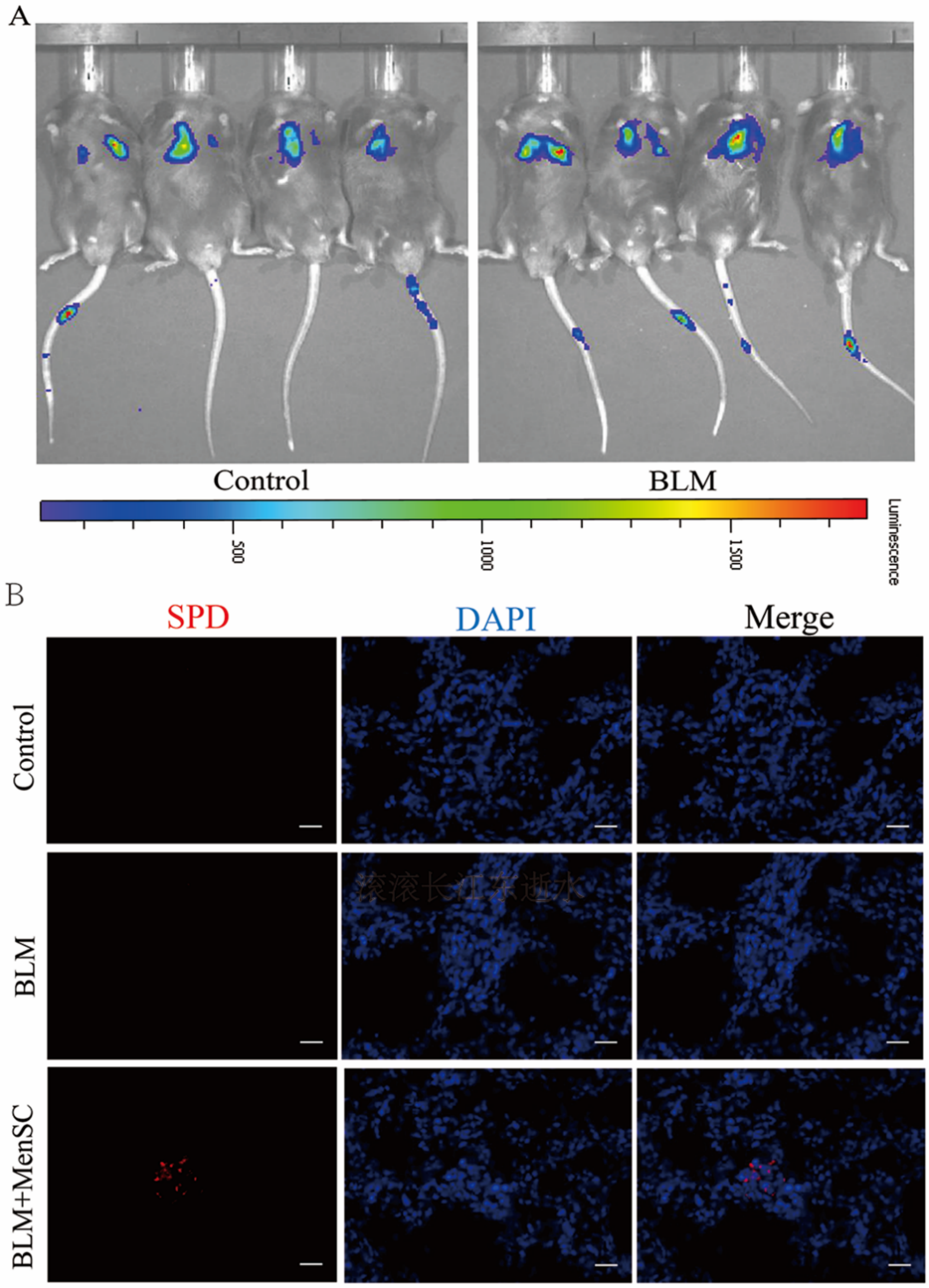

Figure S1. Migration of MenSCs and differentiation of MenSCs in vivo. A Migration of MenSCs in the lung of mice observed by a living imaging system (n=4). **B** surface protein D expression examined in the fibrotic lungs of mice (n=4). Scale bar: 20μm.
