# Supplementary material for: Human menstrual blood-derived stem cells mitigate bleomycin-induced pulmonary fibrosis through anti-apoptosis and anti-inflammatory effects
Source: Stem Cell Res Ther. 2020 Nov 11;11:477. doi: 10.1186/s13287-020-01926-x (PMC7656201; doi:10.1186/s13287-020-01926-x)
Supplement: Supplementary file 2 — Additional file 2. [file 13287_2020_1926_MOESM2_ESM.pdf]

Additional file 2

Supplementary figure 2

QAM-CYT-4

	1 2 3 4	1 2 3 4	1 2 3 4
A	POS1	POS2	Amphiregulin
B	Axl	CD27 Ligand	CD30 (TNFRSF8)
C	CD40 (TNFRSF5)	CXCL16	EGF
D	E-Selectin	Fractalkine	GITR (TNFRSF18)
E	HGF	IGFBP-2	IGFBP-3
F	IGFBP-5	IGFBP-6	IGF-1
G	IL-12 p70	IL-17E (IL-25)	IL-17F
H	IL-1 ra (IL-1 F3)	IL-2 R alpha	IL-20
I	IL-23 p19	IL-28A	I-TAC (CXCL11)
J	MDC (CCL22)	MIP-2	MIP-3 alpha (CCL20)
K	Osteopontin (SPP1)	Osteoprotegerin	Prolactin
L	Pro-MMP-9	P-Selectin	Resistin
M	SCF	SDF-1 alpha	Thrombopoietin (TPO)
N	VCAM-1 (CD106)	VEGF-A	VEGF-D

QAM-CYT-5

	1 2 3 4	1 2 3 4	1 2 3 4
A	POS1	POS2	bFGF
B	BLC (CXCL13)	CD30 Ligand	Eotaxin-1 (CCL11)
C	Eotaxin-2 (MPIF-2)	Fas Ligand (TNFSF6)	GCSF
D	GM-CSF	ICAM-1 (CD54)	IFN-gamma
E	IL-1 alpha	IL-1 beta	IL-2
F	IL-3	IL-4	IL-5
G	IL-6	IL-7	IL-10
H	IL-12 p40	IL-13	IL-15
I	IL-17A	IL-21	KC (CXCL1)
J	Leptin	LIX	MCP-1 (CCL2)
K	MCP-5	M-CSF	MIG (CXCL9)
L	MIP-1 alpha (CCL3)	MIP-1 gamma	Platelet Factor 4
M	RANTES (CCL5)	TARC (CCL17)	I-309 (CCL1)
N	TNF RI (TNFRSF1A)	TNF RII (TNFRSF1B)	TNF-alpha

QAM-CYT-6

	1 2 3 4	1 2 3 4	1 2 3 4
A	POS1	POS2	4-1BB (CD137)
B	Ace	ALK-1	Cardiotriohin-1 (CT-1)
C	CD27 (TNFRSF7)	CD40 Ligand	CTLA-4 (CD152)
D	Decorin	DKK-1	Dtk
E	Endoglin (CD105)	Fc-gamma-RIIB	Flt-3 Ligand
F	Galectin-1	Galectin-3	Gas 1
G	Gas 6	GITR Ligand	HAI-1
H	HGFR	IL-1 R4 (ST2)	IL-3 R beta
I	IL-9	JAM-A (CD321)	Leptin R
J	L-selectin (CD62L)	Lymphotactin	MadCAM-1
K	MFG-E8	MIP-3 beta (CCL19)	Neprilysin
L	Pentraxin-3 (TSG-14)	RAGE	TACI
M	TREM-1	TROY	TSLP
N	TWEAK R	VEGF R1	VEGF R3

QAM-CYT-7

	1 2 3 4	1 2 3 4	1 2 3 4
A	POS1	POS2	CD80 (B7-1)
B	BAFF R	Betacellulin (BTC)	C5a
C	CCL6	CD48 (SLAMF2)	CD6
D	Chemerin	Clusterin	CXCL15
E	Cystatin C	DAN	DLL4
F	EDAR	Endocan	Fetuin A
G	H60	IL-33	IL-7 R alpha
H	Kremen-1	Limitin	Lipocalin-2 (NGAL)
I	LOX-1	Marapsin	MBL-2
J	Meteorin	Nope	NOV (CCN3)
K	Osteoactivin	OX40 Ligand	P-Cadherin
L	Periostin	PIGF-2	Progranulin
M	Prostasin	Renin 1	Testican 3
N	TIM-1 (KIM-1)	TRAIL (TNFSF10)	Tryptase epsilon

QAM-CYT-8

	1 2 3 4	1 2 3 4	1 2 3 4
A	POS1	POS2	6Ckine
B	Activin A	ADAMTS1	Adiponectin
C	ANG-3	ANGPTL3	Artemin
D	CCL28	CD36	Chordin
E	CRP	E-Cadherin	Epigen
F	Epiregulin	Fas	Galectin-7
G	gp130	Granzyme B	Gremlin
H	IFN-γ R1	IL-17B	IL-17B R
I	IL-22	MIP-1β	MMP-2
J	MMP-3	MMP-10	PDGF-AA
K	Persephin	sFRP-3	Shh-N
L	SLAM	TCK-1	TECK
M	TGFβ1	TRANCE	TremL1
N	TWEAK	VEGF-B	VEGF-R2

Figure S2. Two hundred mouse cytokines detected by antibody microarrays.
