# Supplementary material for: Human menstrual blood-derived stem cells mitigate bleomycin-induced pulmonary fibrosis through anti-apoptosis and anti-inflammatory effects
Source: Stem Cell Res Ther. 2020 Nov 11;11:477. doi: 10.1186/s13287-020-01926-x (PMC7656201; doi:10.1186/s13287-020-01926-x)
Supplement: Supplementary file 3 — Additional file 3. [file 13287_2020_1926_MOESM3_ESM.pdf]

Additional file 3

Supplementary figure 3

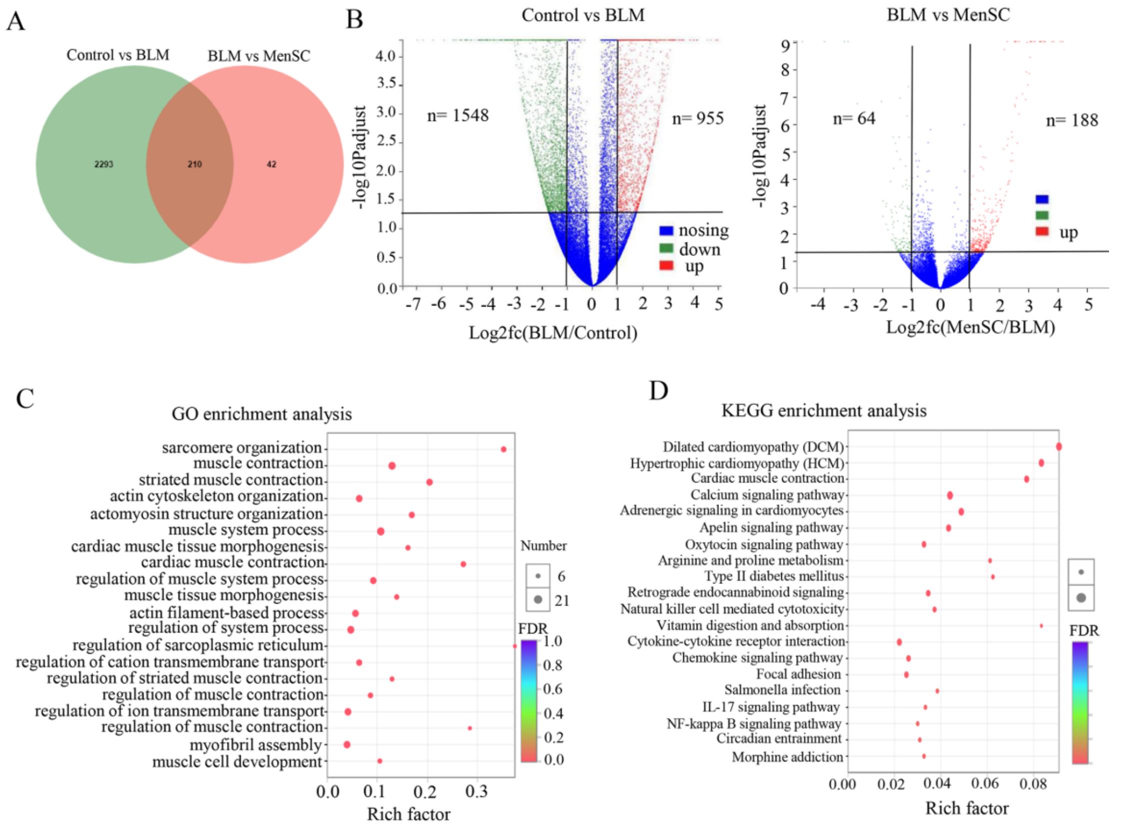

Figure S3. RNA-sequence of lung tissues in three different groups. A Veen analysis between two different groups (n=5). **B** A volcano plot showed different gene. Red, green, blue color represents upregulated, downregulated, and unchanged expression, respectively. **(C, D)**: Top 20 significantly different results from GO (C) and KEGG pathway (D) analyses.
