# Supplementary material for: Human menstrual blood-derived stem cells mitigate bleomycin-induced pulmonary fibrosis through anti-apoptosis and anti-inflammatory effects
Source: Stem Cell Res Ther. 2020 Nov 11;11:477. doi: 10.1186/s13287-020-01926-x (PMC7656201; doi:10.1186/s13287-020-01926-x)
Supplement: Supplementary file 4 — Additional file 4. [file 13287_2020_1926_MOESM4_ESM.pdf]

Additional file 4

Supplementary figure 4

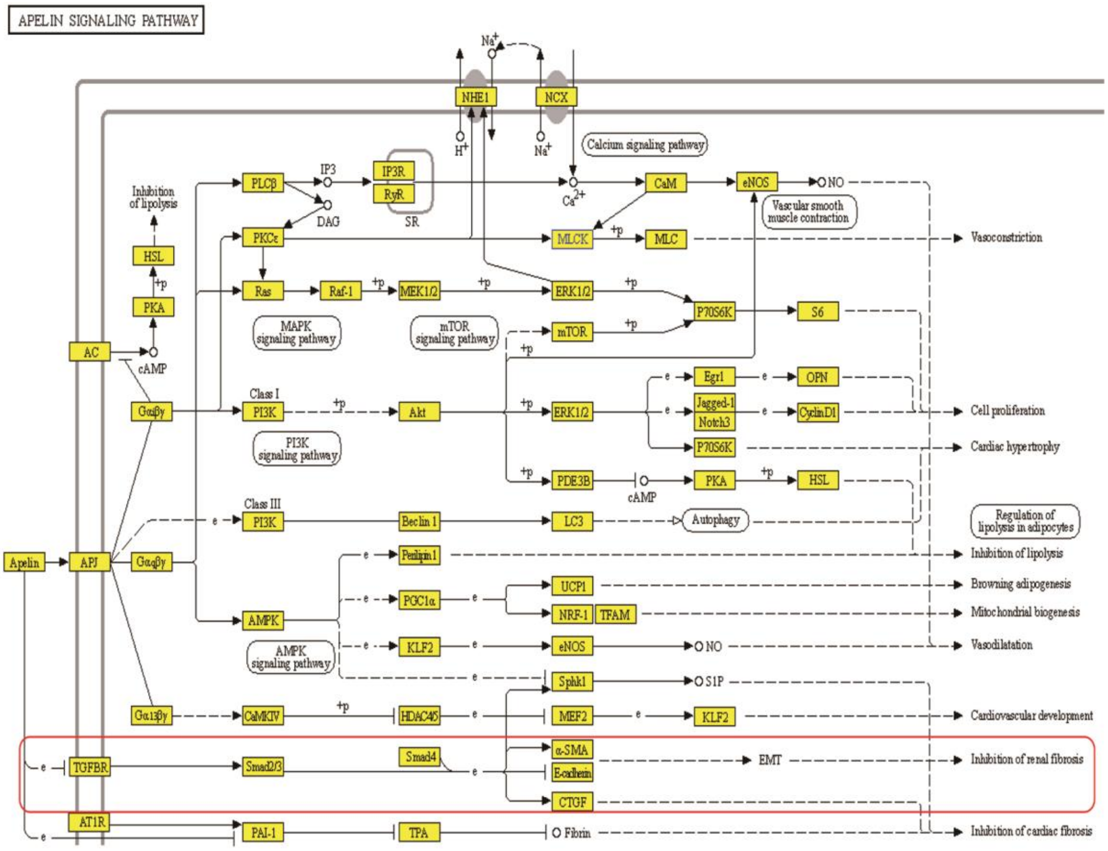

Figure S4. Bioinformatics analysis of the Apelin signaling pathway: expression of a-SMA, CTGF and E-cadherin related to renal fibrosis.
