# Supplementary material for: Human menstrual blood-derived stem cells mitigate bleomycin-induced pulmonary fibrosis through anti-apoptosis and anti-inflammatory effects
Source: Stem Cell Res Ther. 2020 Nov 11;11:477. doi: 10.1186/s13287-020-01926-x (PMC7656201; doi:10.1186/s13287-020-01926-x)
Supplement: Supplementary file 5 — Additional file 5. [file 13287_2020_1926_MOESM5_ESM.pdf]

Additional file 5

Supplemental table 1

Table S1. Anti-body list Related to figure 1. 4. 7 and figure S1.

Anti-body Name	Manufacturer	Cat. Num.	Application	Dilution ratio/Dosage
PE-mouse anti-human CD29	BD Biosciences	561795	Flow Cyt	10μl
PE-mouse anti-human CD34	BD Biosciences	560941	Flow Cyt	10μl
PE-mouse anti-human CD45	BD Biosciences	560975	Flow Cyt	10μl
PE-mouse anti-human CD73	BD Biosciences	561014	Flow Cyt	10μl
PE-mouse anti-human CD90	BD Biosciences	561970	Flow Cyt	2.5μl
PE-mouse anti-human CD105	BD Biosciences	560839	Flow Cyt	2.5μl
PE-mouse117 anti-human CD	BD Biosciences	561682	Flow Cyt	2.5μl
PE-mouse anti-human HLA-DR	BD Biosciences	560943	Flow Cyt	10μl
PE mouse IgG1	BD Biosciences	555749	Flow Cyt	10μl
PE mouse IgG2a	BD Biosciences	555574	Flow Cyt	10μl
TGF-β1	Abcam	ab92486	WB/IHC-P	1:1000/1:100

a-SMA	Abcam	ab5694	WB/IHC-P	1:500/1:100
CTGF	Abcam	ab6992	WB/IHC-P	1:1000/1:200
E-cadherin	Abcam	ab760555	WB/IHC-P	1:1000/1:100
Fibronectin	Abcam	ab2413	WB/IHC-P	1:1000/1:100
Pro-SPC	Abcam	ab90716	IHC-P	1:100
P-Smad3	Abcam	ab52903	WB	1:2000
Smad3	Abcam	ab208182	WB	1:1000
Bcl2	Cell Signaling	2876S	WB	1:1000
BAX	Cell Signaling	2772	WB	1:1000
Cleaved caspase3	Cell Signaling	9664	WB	1:1000
GAPDH	Abcam	ab8245	WB	1:5000
β-actin	Abcam	ab8226	WB	1:5000
SPD	Santa cruz	Sc-13979	IF/ICC	1:100
N-cadherin	Abcam	ab18203	ICC	1:50
Collagen1	Abcam	Ab260043	ICC	1:250
APC/CY7-CD45	BD Bioscience	557659	Flow Cyt	5μg/10^^{6}cell
APC/CY7-CD31	Biolegend	102439	Flow Cyt	0.5μg/10^^{6} cell

BV711-CD326	BD Bioscience	563134	Flow Cyt	5μg/10^^{6}cell
CD16/32	BD Bioscience	553142	Flow Cyt	1μg/10^^{6}cell
FVS700	BD Bioscience	564997	Flow Cyt	0.32μg/10^^{6}cell
Alexa Fluo-488-conjugated goat anti-mouse	Abcam	ab150117	ICC	1:500
Alexa Fluo-633-conjucated goat anti-rabbit	Invitrogen	A-21071	ICC	1:500
