# Supplementary material for: Human menstrual blood-derived stem cells mitigate bleomycin-induced pulmonary fibrosis through anti-apoptosis and anti-inflammatory effects
Source: Stem Cell Res Ther. 2020 Nov 11;11:477. doi: 10.1186/s13287-020-01926-x (PMC7656201; doi:10.1186/s13287-020-01926-x)
Supplement: Supplementary file 6 — Additional file 6. [file 13287_2020_1926_MOESM6_ESM.pdf]

Additional file 6

Supplemental table 2

**Table S2. Primer list related to Figure 3.**

| Gene name | Primer name | Species |
|---|---|---|
| α-SMA | α-SMA-F: AAGAGGAAGACAGCACAGCTC | Mouse |
| | α-SMA-R: GATGGATGGGAAAACAGCC | |
| Fibronectin | fibronectin-F: CTCAGCTTCCGACACCGT, | Mouse |
| | fibronectin-R: CAGGCGCTGTTGTTTGTG | |
| Collagen1 | Collagen1-F: GCTCCTCTTAGGGGCCACT, | Mouse |
| | Collagen1-R: CCACGTCTCACCATTGGGG | |
| GAPDH | GAPDH-F: AGGTCGGTGTGAACGGATTTG | Mouse |
| | GAPDH-R: TGTAGACCATGTAGTTGAGGTCA | |
