# Supplementary material for: Human menstrual blood-derived stem cells mitigate bleomycin-induced pulmonary fibrosis through anti-apoptosis and anti-inflammatory effects
Source: Stem Cell Res Ther. 2020 Nov 11;11:477. doi: 10.1186/s13287-020-01926-x (PMC7656201; doi:10.1186/s13287-020-01926-x)
Supplement: Supplementary file 7 — Additional file 7. [file 13287_2020_1926_MOESM7_ESM.pdf]

中国典型培养物保藏中心

CHINA CENTER FOR TYPE CULTURE COLLECTION (CCTCC)

Wuhan University, Wuhan 430072, China

Phone: 86-027-68752093 **Fax:** 86-027-68754833 **Email:** shenchao@whu.edu.cn

05-24-2018

Entrusted by Zhejiang University, CCTCC has conducted identification experiments on the MenSC cell line, and come to the following conclusions:

1. There was no third allele found in all the locations of MenSC cell line, it indicating that there was no cross-contaminant of human source cell line.
2. Compared the STR data of MenSC cell line in the databases of ATCC and DSMZ, its profile does not exactly match with any of the current data (Table 1).
3. The STR data of MenSC cell line and NCI-H64 (CRL-5976) cell line matches the highest rate of 75% in ATCC databases.

Manager：

China Center for Type Culture Collection

Note:

1. The result is only responsible for the test sample, and the genomic DNA will be reserved for three month.
2. Reference of human cell line authentication: ANSI/ATCC ASN-0002-2011

Table 1. The alleles of 21 locations in MenSC cell line

MenSC cell line（Fig. No.XB7641）		
D19S433	13	14.2
D5S818	11	11
D21S11	29	30
D18S51	13	17
D6S1043	12	13
AMEL	X	X
D3S1358	15	16
D13S317	10	10
D7S820	8	10
D16S539	10	12
CSF1PO	9	10
Penta D	9	12
D2S441	10	12
vWA	17	18
D8S1179	14	14
TPOX	8	11
Penta E	18	19
TH01	9	9
D12S391	20	21
D2S1338	18	24
FGA	21	21
FGA	22	22

CCTC

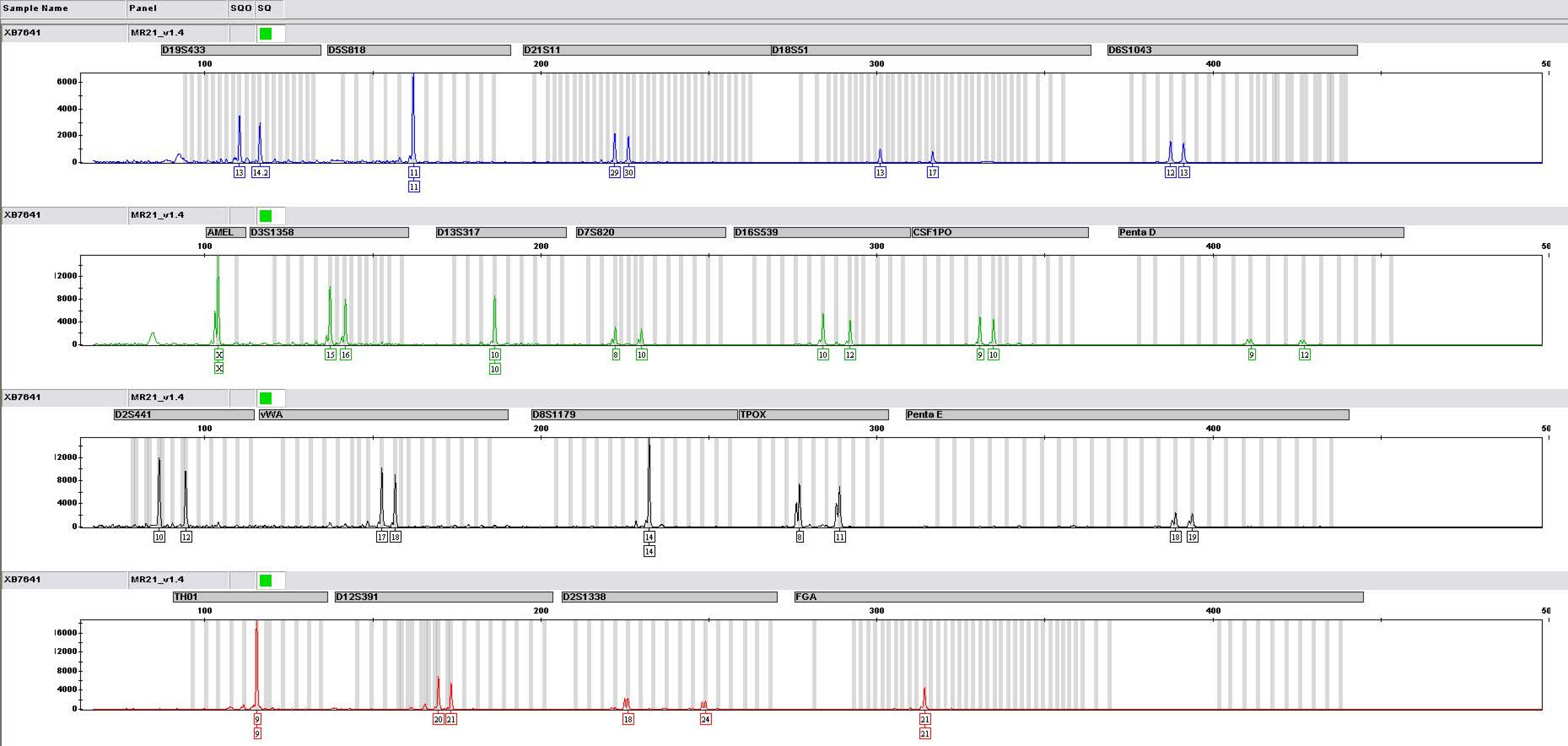

Sample Name
Panel
SQO
SQ
XB7641
MR21_v1.4
D19S433
D5S818
D21S11
D18S51
D6S1043
13 14.2
11 11
29 30
13 17
12 13
AMEL
D3S1358
D13S317
D7S820
D16S539
CSF1PO
Penta D
X X
15 16
10 10
8 10
10 12
9 10
9 12
D2S441
vWA
D8S1179
TPOX
Penta E
10 12
17 18
14 14
8 11
18 19
TH01
D12S391
D2S1338
FGA
9 9
20 21
18
24
21 21
